# The utility of wastewater surveillance for monitoring SARS-CoV-2 prevalence

**DOI:** 10.1093/pnasnexus/pgae438

**Published:** 2024-10-04

**Authors:** Cathal Mills, Marc Chadeau-Hyam, Paul Elliott, Christl A Donnelly

**Affiliations:** Department of Statistics, University of Oxford, Oxford OX1 3LB, United Kingdom; Pandemic Sciences Institute, University of Oxford, Oxford OX3 7DQ, United Kingdom; School of Public Health, Imperial College London, White City Campus, London W12 0BZ, United Kingdom; School of Public Health, Imperial College London, White City Campus, London W12 0BZ, United Kingdom; Department of Statistics, University of Oxford, Oxford OX1 3LB, United Kingdom; Pandemic Sciences Institute, University of Oxford, Oxford OX3 7DQ, United Kingdom; School of Public Health, Imperial College London, White City Campus, London W12 0BZ, United Kingdom

**Keywords:** COVID-19, Omicron SARS-CoV-2, vaccination, shedding rates, wastewater-based epidemiology

## Abstract

Public health authorities have increasingly used wastewater-based epidemiology (WBE) to monitor community transmission of SARS-CoV-2 and other agents. In this study, we evaluate the utility of WBE during the COVID-19 pandemic in England for estimating SARS-CoV-2 prevalence. We use wastewater data from the Environmental Monitoring for Health Protection program and prevalence data from the REal-time Assessment of Community Transmission-1 study. Across the pandemic, we describe how wastewater-based modeling can achieve representative SARS-CoV-2 prevalence estimates in fine and coarse spatial resolutions for relatively short-time horizons (of up to 1 month), and thus assist in filling temporal gaps in surveillance. We infer a temporally evolving relationship between wastewater and prevalence which may limit the utility of WBE for estimating SARS-CoV-2 prevalence over longer time horizons without a concurrent prevalence survey. Exploring further our finding of time-varying, population-level fecal shedding, we characterize WBE for SARS-CoV-2 prevalence as (i) vaccination coverage dependent and (ii) variant- specific. Our research suggests that these factors are important considerations in future uses of WBE by public health authorities in infectious disease outbreaks. We further demonstrate that WBE can improve both the cost efficiency and accuracy of community prevalence surveys which on their own may have incomplete geographic coverage and/or small sample sizes. Therefore, in England, for the objective of high spatial resolution prevalence monitoring, strategic use of SARS-CoV-2 wastewater concentration data nationally could have enhanced, but not replaced, community prevalence survey programs.

**Significance Statement**

Wastewater-based epidemiology (WBE) was employed globally throughout the COVID-19 pandemic, including for the estimation of clinical indicators. However, despite recent advances, the dynamics of the relationship between SARS-CoV-2 wastewater concentrations and community prevalence remains an underdeveloped area of research. Likewise, it remains unclear how, and if, WBE could be employed effectively for monitoring prevalence across a nation over sustained periods of time. Here, we discover moderate-to-strong potential for wastewater-model-based estimation of SARS-CoV-2 community prevalence yet also find a temporally evolving prevalence-to-wastewater relationship in the aftermath of widespread vaccination and changing dominant variants. These two factors of vaccination and variants influence the time horizon for which reliable wastewater-based prevalence estimates can be made without a concurrent community prevalence survey, for both high and coarse spatial resolutions. Finally, we demonstrate that despite the temporally evolving relationship, SARS-CoV-2 wastewater surveillance could enable greater logistical and economic efficiencies for high-resolution community prevalence surveys, although wastewater concentration data alone, in the absence of a calibrating prevalence survey, is likely insufficient for reliable, stable estimation of SARS-CoV-2 prevalence over long-time horizons.

## Introduction

Monitoring the distribution of viruses has been a common use of wastewater-based epidemiology (WBE) in recent years (1). WBE involves collection of samples from wastewater sites that capture human excretions through urine or fecal matter, and has enabled, for example, surveillance of poliovir uses (2). During the COVID-19 pandemic, as governments and public health authorities around the world sought to monitor the transmission and spread of SARS-CoV-2 (the virus that causes COVID-19), WBE was applied as an infection surveillance tool, since SARS-CoV-2 is excreted in the feces of both symptomatic and asymptomatic infected individuals.

Clinically confirmed SARS-CoV-2 prevalence estimates are sensitive to test-seeking biases, asymptomatic infections, and clinical testing capacity. Conversely, WBE represents an indirect, noninvasive, population-level infection surveillance tool for cost-effective, real-time monitoring of pathogen transmission (3, 4). Several studies have established strong correlations between wastewater SARS-CoV-2 concentrations and reported COVID-19 cases (5–7), while others estimated the time dependency and lead time (varying from 4 to 10 days) from detection in wastewater to date of testing in clinical cases (8–10). During the pandemic, SARS-CoV-2 prevalence in England was estimated for 45 sewage site catchments (11), while further research estimated weekly viral wastewater concentrations in a spatially continuous domain (12). More recently, the issue of how best to incorporate wastewater surveillance into a prevalence estimation framework has been explored with a specific focus on now casting community prevalence in counterfactual scenarios (13).

Here, across 21 months of the COVID-19 pandemic in England, we provide a high-resolution spatiotemporal analysis and evidence synthesis of the utility of WBE for estimating community SARS-CoV-2 prevalence. Our analysis uses data from one of the world's largest community prevalence surveys; the REal-time Assessment of Community Transmission-1 (REACT-1) study, and the Environmental Monitoring for Health Protection (EMHP) wastewater surveillance program.

Our geospatial framework maps wastewater concentrations from a sewage treatment plant to the level of a Lower Tier Local Authority (LTLA). Then, our analysis provides a rigorous investigation of the relationship between wastewater concentration estimates and estimated infection prevalence from REACT-1. We investigate the stationarity of this relationship over time and whether we can disentangle factors that influence the prevalence-to-wastewater relationship. Leaning on this exploratory analysis, we perform extensive modeling analyses to investigate the spatial and temporal extent to which WBE can facilitate reliable estimation of community prevalence of SARS-CoV-2. Our analyses and the lessons learned therein will serve as recommendations for future infectious disease outbreaks where authorities wish to combine infection surveillance by survey data and WBE.

## Results

Our analysis, across two distinct studied periods of the COVID-19 pandemic (labeled *early* and *late* periods which are segmented due to surveillance differences—see Materials and methods), focuses on how SARS-CoV-2 wastewater concentration estimates can be associated with the community SARS-CoV-2 prevalence estimates from individual rounds of the REACT-1 survey. We consider varying spatial scales in our analyses; from coarser (regional) to fine (LTLA) resolution. Our wastewater-based, gradient boosting model learned the relationship between wastewater concentrations and survey prevalence estimates during model training. The wastewater-based model was then used to estimate out-of-sample (i.e. hypothetically unobserved) SARS-CoV-2 prevalence within individual prevalence survey rounds. For context, the aforementioned *early* period of analysis (rounds 3 to 11, from 2020 July 24 to 2021 May 3) was the first period of overlap between the wastewater surveillance program and REACT-1 survey. The period corresponded to a duration of relatively low, stable SARS-CoV-2 prevalence levels (mean LTLA estimate: 0.55%) and vaccination was at low levels (mean fully vaccinated proportion for an LTLA in round 11 was 12.2%). Conversely, the 10-month *late* period (rounds 12–19, 2021 May 20 to 2022 March 31) incorporated expanded wastewater testing coverage and the epidemic's evolution was characterized by high prevalence levels, heterogeneous vaccination of the population, and occurrence of a new, highly transmissible Omicron variant with different properties, such as lower viral loads, selective reduction in Omicron infectivity in the intestinal tract, and shorter duration of respiratory shedding, compared with the Delta variant (14–16).

### Relationship between SARS-CoV-2 RNA wastewater concentrations and prevalence

**Fig. 1.**
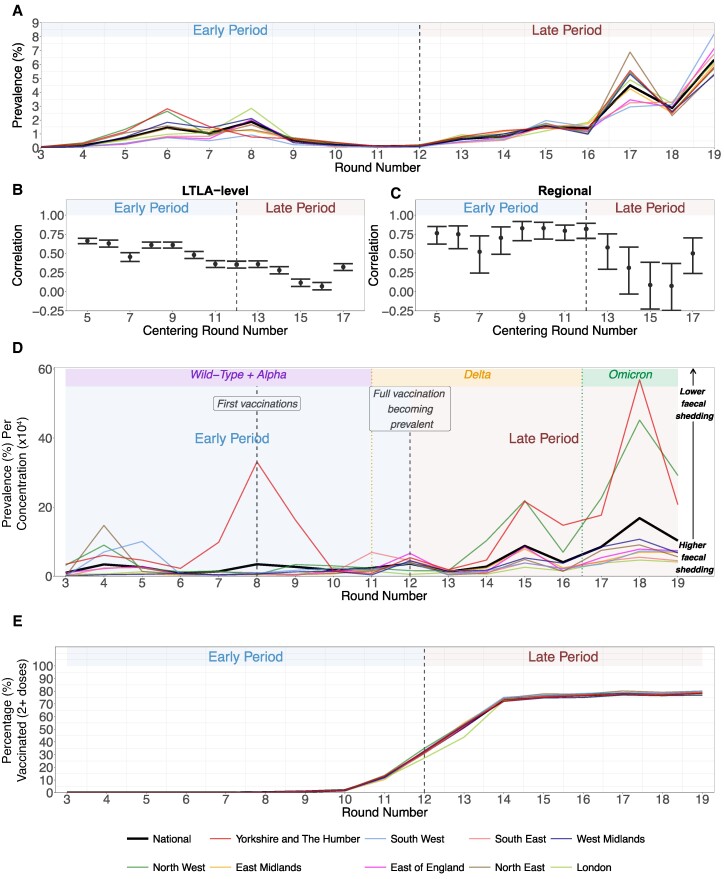
Prevalence–wastewater relationship. A) Population-weighted regional (and national) averages of SARS-CoV-2 prevalence estimates per survey round of the REACT-1 study. B) Spearman's correlation between all LTLA-level observations of SARS-CoV-2 wastewater concentrations and REACT-1 SARS-CoV-2 prevalence across moving five-round windows centered on each survey round from REACT-1 rounds 5 (commencing 2020 September 18) to 17 (ending 2022 January 20). 95% CIs were obtained by the Clopper–Pearson method (17). The number of observations used for computing the correlations across each five-round window varied depending on the number of LTLAs covered by the wastewater surveillance program. C) Analogous visualization of correlation between all observations of regional wastewater concentration and SARS-CoV-2 prevalence estimates within the same moving five-round windows (*n* = 45 for each window). Regional estimates were derived by population-level weighting of the corresponding LTLA-level wastewater concentrations and prevalence estimates. To visualize how any LTLA- and region-specific heterogeneities in wastewater concentrations and/or prevalence estimates impact their individual prevalence–wastewater relationships, we also calculated correlations within each LTLA (region) separately (visualized in Fig. S1). D) Population-weighted regional (and national) REACT-1 prevalence per population-weighted regional (and national) estimated wastewater concentration by round of data collection, from REACT-1 rounds 3 (2020 July 24) to 19 (2022 March 31). Greater values of the ratio correspond to lower implied population-level fecal shedding rates, i.e. per positive infected individual. For reference, vaccination began in round 8 (2021 January 6) with full vaccination becoming prevalent from round 12 (2021 May 20) onwards (when the survey round estimate of the national average proportion of the entire population fully vaccinated is 31.7%), the Delta variant was dominant from rounds 11 to 16 (2021 April 15 to 2021 December 14), and the Omicron BA.1 and BA.2 subvariants are dominant in rounds 17 to 19 (from 2022 January 5 to 2022 March 31). E) Population-weighted regional (and national) proportions of the population with two or more doses of a licensed vaccine, as estimated per survey round of the REACT-1 study. Note that only data from REACT-1 rounds 3 to 19 are displayed as this represents the time period which overlaps with the wastewater surveillance program.

Before assessing how a wastewater-based model may be used to estimate SARS-CoV-2 prevalence, we describe the relationship between SARS-CoV-2 prevalence estimates and corresponding wastewater concentrations, at varying time periods throughout the COVID-19 pandemic. Consistent with the underlying scientific premise, in the *early* period (REACT-1 rounds 3 to 11, from 2020 July 24 to 2021 May 3), estimated wastewater concentrations at an LTLA level were moderately to strongly correlate with SARS-CoV-2 prevalence (Spearman's correlation *r=* 0.62; 95% CI: 0.59–0.65). These trends were observed at fine spatial resolution (LTLA), and stronger relationships were observed at coarser spatial resolution between (population-weighted) regional and national average wastewater concentration and corresponding (population-weighted) averages of prevalence estimates (Table S1). Looking at the time evolution of the prevalence–wastewater relationships (Fig. 1), as measured by correlations between all wastewater concentrations and SARS-CoV-2 prevalence within moving (centrally aligned) windows of five survey rounds, we estimated relatively stable, consistently strong relationships until the emergence of the Delta variant and the onset of increasingly widespread vaccination in the population (from rounds 11 to 12 onwards—which began on 2021 April 15 and 2021 May 20, respectively). Indeed, across the entire *late* studied period (rounds 12 to 19, from 2021 May 20 to 2022 March 31), we found the relationship between concentrations and prevalence to be more complex, volatile, and temporally evolving (Fig. 1). For example, within a subset of five pre-Omicron rounds (rounds 12 to 16, 2021 May 20 to 2021 December 14) of the *late* period, we report weaker correlation (*r=* 0.28; 95% CI: 0.23–0.33) between LTLA-level concentrations and prevalence. Similarly diminished relationships were observed between the regional population-weighted prevalence estimates and corresponding wastewater concentrations, despite the increased geographic coverage of the wastewater surveillance program during this period. We note that these trends of diminishing correspondence were similarly observed if we assessed the correlations (over time) between prevalence and concentration estimates within each LTLA (or region) separately (Fig. S1).

To further investigate these findings of time-varying complexities, we propose here a new metric (which may be a useful tool for future infectious disease outbreaks)—the estimated prevalence per concentration in wastewater (Fig. 1D). The metric captures the prevalence-to-wastewater relationship, and so population-level fecal shedding over time (with higher values indicating lower average fecal shedding per person). Relationship complexity (suggested previously by the aforementioned five-round-windowed correlations and now in the prevalence per concentration) was evident in the presence of (i) rapid rollout of vaccination of LTLA populations nationally, and (ii) rapid replacement of the Delta variant by the Omicron BA.1 and BA.2 subvariants (Fig. 1). In terms of the impacts of vaccination on the prevalence-to-wastewater relationship, we observed substantially diminishing correlation between prevalence estimates and wastewater concentrations (Fig. 1) during five-round windows where full vaccination was increasingly prevalent (i.e. from rounds 12 onwards). In terms of our proposed metric, across all observations from rounds 12 to 19 (from 2021 May 20 to 2022 March 31), the estimated prevalence per wastewater concentration was moderately correlated (*r* = 0.45; 95% CI: 0.41–0.48) with the corresponding (estimated) proportion of an LTLA population that was fully vaccinated (two or more doses of any vaccine), though correlation was stronger when we instead considered the population proportion fully vaccinated in the previous round (*r* = 0.51; 95% CI: 0.48–0.54). Similarly, focusing on the evolution of vaccination prevalence per concentration over time within LTLAs, the median of the LTLAs’ correlations (*n* = 309), calculated using each LTLA's observations across eight survey rounds, between prevalence per concentration and fully vaccinated proportion from the previous round was 0.64.

Thus, in understanding the association between SARS-CoV-2 prevalence and wastewater concentrations, it is important to account for the proportion of a population fully vaccinated and specifically the percentage of vaccinated individuals in the preceding month. The implied lag may reflect time taken for a vaccine to become effective at reducing fecal shedding. Inclusion of the vaccination–concentration interaction (see Table S2) increased the wastewater-infection prevalence correlation (*r* = 0.71, 95% CI: 0.69–0.73) relative to the unadjusted model.

By mid-December 2021, the REACT-1 study and EMHP wastewater program had found that Omicron BA.1 had become the dominant SARS-CoV-2 variant in England (18, 19). Here, we show that the percentage testing positive per concentration in wastewater increased during the Omicron period, indicating less fecal shedding per infected individual at a population level (Fig. 1). Reduced levels of detected population-level fecal shedding were observed as wastewater concentrations did not show the same magnitude of increases as prevalence levels. Such reduced population-level shedding was observed during survey rounds and there was initially a breakdown in the longitudinal correspondence between LTLA-level concentration and prevalence (around REACT-1 round 17), although the correlation strengthens when we narrow our focus to this new period with widespread, reduced population-level fecal shedding (Figs. 1 and S1).

### Out-of-sample wastewater-model-based estimates of REACT-1 prevalence

Moving toward our wastewater-based modeling analysis, we now describe results in out-of-sample environments, where estimates were out-of-sample in the sense that we omitted the prevalence data for each (entire) individual testing survey round (and all subsequent rounds). To assess the predictive performance of our (wastewater-based) models in estimating the prevalence, our out-of-sample approach split the data into training and testing sets. We considered two data splitting strategies: (i) iterative estimates where we estimated community SARS-CoV-2 prevalence within a single survey (testing) round using a wastewater-based model trained/calibrated on all prevalence data up to one round before each testing round, and (ii) multistep estimates where we estimated prevalence for three individual (testing) survey rounds using a wastewater-based model where the model is not recalibrated across the three testing rounds.

**Fig. 2.**
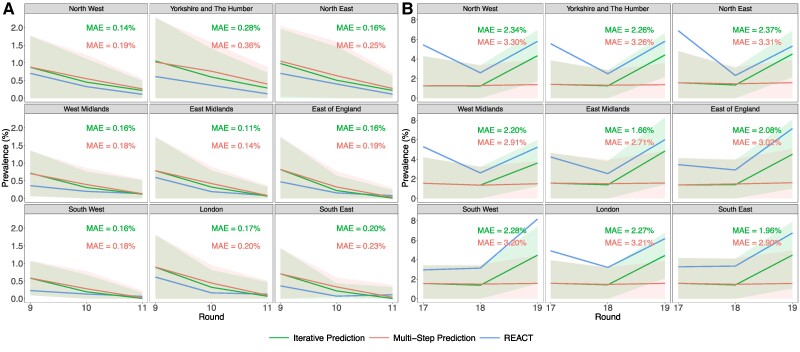
Regional out-of-sample, wastewater-model-based prevalence estimates in A) rounds 9 to 11 (2021 February 4 to 2021 May 3) and B) rounds 17 to 19 (2022 January 5 to 2022 March 31). Out-of-sample wastewater-model-based prevalence estimates are shown alongside REACT-1 prevalence (blue), where estimates were out of sample in the sense that we omitted the prevalence data for each (entire) individual testing survey round (and all subsequent rounds). The wastewater-based model was trained at an LTLA level (i) with iterative (green) updating of the model (thus expanding the training set every round with an additional training round), and (ii) without model recalibration (red—which means there was a fixed training set size across the three testing rounds). The two scenarios amount to employing a wastewater-based model for either a single survey round (i.e. a relatively short period of time up to 1 month) or for three survey rounds (i.e. a more sustained period of up to 3 months), and thus enable comparison of predictive performance with, and without, regular model calibration of the wastewater-to-prevalence relationship. The wastewater-model-based and REACT-1 prevalence estimates for each region (per individual round) were obtained by weighting the LTLA-level prevalence estimates by the corresponding LTLA populations within the region. The shaded 95% PIs were obtained by 5,000 nonparametric bootstrap samples. In A), the mean regional (population-weighted) average prevalence across rounds 9 to 11 was 0.28%, and in B), the mean regional (population-weighted) average prevalence across rounds 17 to 19 was 4.57%. The substantial differences in prevalence levels are the reason for the different *y*-axes for prevalence across A) and B).

Employing an iteratively updating wastewater-based, gradient boosting model, in the *early* period, from REACT-1 rounds 7 to 11 (2020 November 3 to 2021 May 3), we report weak-to-moderate within-round predictive accuracy for estimating LTLA-level REACT-1 prevalence, which is reflected in Mean Absolute Error (MAE) values, occasional failure to detect which LTLAs were experiencing the most extreme prevalence levels across the nation, and suboptimal accuracy for detection of prevalence level changes (Table S3). These results are reflective of modest predictive abilities of the wastewater-based model, yet also collectively suggest occasional difficulties in relying exclusively on a wastewater model (post-calibration) for high-resolution prevalence inferences. Nevertheless, if we assess the iteratively updating wastewater-based model's out-of-sample estimates collectively (i.e. over time) across the five survey rounds, there was moderate-to-strong predictive accuracy (MAE = 0.37%) and strong correspondence between the model's estimates and the survey prevalence estimates, both within LTLAs’ individual time series (median *r* = 0.85) and across all LTLA-level observations (*r* = 0.66). Moving toward a coarser regional geographic scale (where population-weighted regional mean prevalence estimates were obtained by weighting the LTLA-level estimates by their corresponding populations), there was greater representativeness in the wastewater-based model's estimates (Figs. 2 and S3, Tables S3 and S4). The superior predictive accuracy is reflected in the ability to reliably estimate regional average prevalence (MAE = 0.27%) and the accuracy of 88.9% (95% CI: 75.9–96.3%) in estimating whether the regions were experiencing increasing or decreasing prevalence levels.

Remaining within the *early* period, but focusing on REACT-1 rounds 9 to 11 (2021 February 4 to 2021 May 3) to enable comparison between the iteratively updated and multistep estimates produced for these three rounds, we observed relatively similar, weak-to-moderate predictive accuracy at an LTLA level for the iteratively updating and multistep, wastewater-based model's estimates (Table S5). At a regional level, both the iteratively updating and multistep wastewater-based model's estimates were strong at estimating prevalence movements (i.e. increasing or decreasing prevalence levels) and the correlation between the model-based estimates and REACT-1 survey-based prevalence was strong in both settings. Nevertheless, the iteratively updating model's (population-weighted) regional prevalence estimates benefited from the recalibrations of the prevalence-to-wastewater relationship as its estimates (*n* = 27) outperformed across the metrics of MAE (iterative: 0.17%, multistep: 0.21%), detecting prevalence movements (iterative: 92.6%, multistep: 77.8%), and correlations with the prevalence (iterative: 0.94, multistep: 0.93; Table S5).

Moving toward the *late* period (2021 May 20 to 2022 March 31) with higher prevalence levels and increasing population vaccination levels, the iteratively updating wastewater-based model's out-of-sample prevalence estimates, at an LTLA level for REACT-1 rounds 15–19 (2021 October 19 to 2022 March 31), indicated moderate predictive power in terms of detecting prevalence movements (75.4%, 95% CI: 73.2–77.5%) and tracking precise prevalence levels (*r* = 0.65; Tables S6 and S7). Similarly, looking at the out-of-sample estimates for the five rounds collectively (i.e. over time), there was once again a moderate-to-strong correspondence between our LTLA-level model-based estimates and survey-based prevalence (*r* = 0.65, MAE = 1.65%) and likewise, when analyzing the correspondence within individual LTLAs separately (median *r* = 0.78, Q1 = 0.57, Q3 = 0.90). Largely similar trends were observed in coarser regional spatial resolution when we weighted our LTLA-level (wastewater-model-based prevalence) estimates by their corresponding populations within the regions (Fig. S4, Tables S6 and S7).

Nevertheless, despite the iteratively updating model's recalibration of the prevalence-to-wastewater relationship every round (i.e. an expanding model training window), the onset of the Omicron BA.1 peak in REACT-1 round 17 (2022 January 5 to 2022 January 20) was largely underestimated and undetected by our wastewater-based model (Table S6 and Fig. 2). The LTLA-level prevalence estimates departed from survey-based prevalence levels (MAE = 2.76% and movement detection accuracy of 62.8%, 95% CI: 57.1–68.2%). Focusing further on the comparison of the iterative updating and multistep test set estimates during the Omicron wave of rounds 17 to 19 (from 2022 January 5 to 2022 March 31), the multistep estimates suffered severely during these rounds, seemingly due to the variant-induced reduction in population-level fecal shedding and thus, the changes to the prevalence-to-wastewater relationship (Figs. 1 and 2). The poor predictive performance of the multistep estimates across rounds 17 to 19 is reflected in the accuracy for detecting LTLA level (52.0%, 95% CI: 48.7–55.3%) and regional prevalence movements (48.1%, 95% CI: 28.7–68.1%; Table S8). Such inability to detect prevalence movements is in contrast to the iteratively updating model's estimates where the accuracy at an LTLA level was 75.9% (95% CI: 73.1–78.7%) and at a regional level was 81.5% (95% CI: 61.9–93.7%).

Despite the inability of our iteratively trained wastewater model to account for large initial surges in REACT-1 prevalence (in round 17) induced by the Omicron variant, following further model calibration in rounds 17 and 18, in round 19 (the Omicron BA.2 peak), our out-of-sample wastewater-based LTLA-level and regional estimates closely track the movements in LTLA level and regional REACT-1 prevalence levels, with corresponding prevalence movement detection accuracies of 91.3% (95% CI: 87.5–94.2%) and 100.0% (95% CI: 66.4–100.0%), respectively (Table S6 and Fig. 2). Indeed, even if there were only a single new model update (or equivalently new calibration round), say round 17 (i.e. omitting REACT-1 round 18), during the Omicron wave (round 19, from 2022 January 5 to 2022 March 31), the wastewater model enabled detection of 87.4% (95% CI: 83.2–90.9%) of the within-round LTLA-level REACT prevalence changes.

### Complementary use of WBE for monitoring SARS-CoV-2 prevalence

We have thus far demonstrated unstable wastewater-based model performance for prevalence estimation in out-of-sample scenarios without concurrent prevalence surveys, possibly due to the temporal inconsistency of the prevalence-to-wastewater relationship (e.g. due to change in fecal shedding related to different variants of the virus) and/or limitations of our spatiotemporal alignment. We now focus on assessing the utility of WBE in England to complement and enhance prevalence surveys for representative, high-resolution estimation of SARS-CoV-2 prevalence for both economic and logistical efficiency. In short, we no longer omit the survey-based prevalence estimates for a full survey round when testing our model's predictive performance as results are now presented for varying simulated (counterfactual) intensities of community prevalence surveys. To ensure robustness of inferences (about the utility of WBE), we focus primarily on results for the *late* period (rounds 12 to 19, 2021 May 20 to 2022 March 31) due to more complete, representative wastewater surveillance. Our approach here is 2-fold as we examine simulated environments (i) with reduced geographic survey coverage, and (ii) with reduced survey-round sample size of participants.

First, for the simulated scenarios of varying LTLA-level geographic survey coverage, across the varying proportions of LTLAs within each round that were used for model training, to enable equitable comparisons, we specified a random 10% of withheld LTLAs from each survey round for wastewater-model-based prevalence estimation. Fixed test set sizes across the training sets of different sizes enabled equitable comparisons of model performance and the procedure was replicated 50 times, across different training–testing environments of the same proportions, to reduce sensitivity of inferences to individual training and testing sets.

As expected, relative to survey-based REACT-1 prevalence levels, maintaining a higher geographic coverage of 224 prevalence surveys throughout the 10-month period allowed more accurate out-of-sample wastewater-based prevalence monitoring across the rounds (Table 1 and Fig. S5). Nevertheless, training environments with fewer LTLA-level observations enabled representative indication of rising or declining REACT-1 prevalence levels for the omitted LTLAs across the sustained period, although the precise estimates tended to depart from survey-based prevalence levels. Similar results were attained for training–testing environments in the *early* period of 2020 July 24 to 2021 May 3 (Table S9), as greater survey geographic coverage (during model training) yielded more accurate wastewater-model-based estimates of test set prevalence, although increasing coverage from 80 to 90% of a REACT-1 round's LTLAs yielded marginally inferior predictive performance (possibly due to incomplete LTLA-level wastewater geographic coverage or the noisiness during epidemic periods with lower SARS-CoV-2 prevalence). The representativeness across rounds of out-of-sample prevalence estimates (over time) for the test set (i.e. omitted) LTLAs, in the presence of a concurrent, reduced-scale prevalence survey, is an apparent consequence of the regular, simultaneous model calibration (which allows for temporal monitoring and thus, updating of the prevalence-to-wastewater relationship). The performance may also be enhanced by the spatial consistency of the prevalence-to-wastewater relationship (Fig. S6). For example, in the *late* period with expanded wastewater surveillance, estimated population-level fecal shedding rates generally became increasingly consistent across LTLAs.

**Table 1.** Results of wastewater-based complementary prevalence estimates across rounds 12 to 19 (2021 May 20 to 2022 March 31) using varying geographic coverage of prevalence surveys for training.

Training–testing	*M*AE	*r*	Change detection (95% CI)	
40–10%	0.69%	0.90	84.2%	(79.7–88.8%)
60–10%	0.63%	0.91	85.4%	(81.0–89.8%)
80–10%	0.59%	0.93	85.8%	(81.5–90.2%)
90–10%	0.56%	0.94	86.6%	(82.4–90.9%)

Wastewater-based models were trained using 40 to 90% of each REACT-1 round's LTLAs (i.e. 40 to 90% of the SARS-CoV-2 prevalence estimates and of the SARS-CoV-2 wastewater concentrations), and 10% of the LTLAs per round were used for real-time, out-of-sample wastewater-based prevalence estimation. Identical test sets of equal size (*n* = 247, across eight rounds) were used (within each training–testing environment) to enable comparison of the model-based information offered by various training environments, while the procedure was repeated 50 times (i.e. 50-folds) to reduce sensitivity to any individual training–testing splits. The average test set prevalence across the 50-folds was 2.25%. Note that MAE represents the median of MAE values and *r* represents the median of the fold-specific Pearson's correlation coefficient across the all testing observations *across* the eight rounds, thus measuring modeling performance across sustained time periods (as opposed to performance within individual survey rounds). *Change Detection* indicates the median of the fold-specific directional accuracy, while the corresponding 95% CI is attained by Clopper–Pearson method, otherwise known as the exact confidence interval (17).

**Fig. 3.**
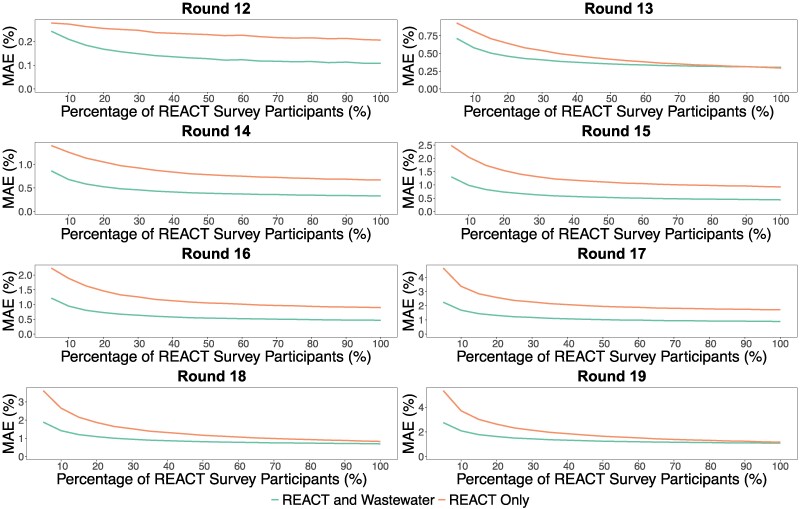
SARS-CoV-2 prevalence estimate accuracy in simulations of reduced survey sample size of participants with and without wastewater modeling. In individual rounds of 12 to 19 (from 2021 May 20 to 2022 March 31), the following procedure was replicated 100 times for proportions ranging from 5 to 100% in increments of 5%: environments were simulated to include 5 to 100% of each LTLA's REACT-1 survey rounds’ total number of participants, and the number of positives was simulated binomially from each such reduced total number of participants with success probability equal to the REACT-1 weighted prevalence. Then, for each proportion, reduced-sized survey-based prevalence levels were used to calibrate wastewater-model-based estimates. Both the reduced-survey-based prevalence levels (orange) and combined reduced survey-wastewater estimates (green) were compared, to a spatially smoothed REACT-1 prevalence benchmark derived from full-sized surveys, via the MAE within each of the 100 simulation replicates. The mean average of the MAE values (across the replicates) is visualized above. Note the different scales of the *y*-axes for MAE which are instructive due to the differences in mean prevalence levels (and hence, MAE) across the eight survey rounds.

Finally, we also evaluated how wastewater surveillance (and our associated modeling) in England could have enabled reductions in the number of participants within survey rounds and thus contributed to greater cost efficiencies in prevalence surveys. Across each setting of varying (simulated) reduced survey-round sample sizes of participants, relative to a (pseudo-independent, ideal) benchmark of spatially smoothed prevalence levels derived from full-sized surveys (see Materials and methods for further details, including limitations), we observed a consistent trend (Fig. 3) of wastewater-model-based estimates improving upon the corresponding reduced-survey-based prevalence estimates (in terms of predictive accuracy). Within each round, the average MAE (relative to the aforementioned benchmark derived from full-sized surveys) expectedly declined as the survey round's sample size totals increased for both the reduced survey only and combined survey-wastewater environments, although the wastewater model consistently yielded improvements upon the survey-based estimates for almost every round and survey sample size of participants. Irrespective of whether we attempted to spatially smooth prevalence estimates from reduced survey total numbers of participants (with and without wastewater), wastewater-based modeling improved the accuracy of prevalence estimates obtained from reduced surveys (where accuracy is defined to the spatially smoothed prevalence benchmark derived from full-sized surveys).

## Discussion

Across a time horizon of 21 months of the COVID-19 pandemic in England, our spatiotemporal analyses are the first nationwide detailed evidence synthesis of (i) the prevalence-to-wastewater relationship, and relatedly, (ii) the utility of WBE for SARS-CoV-2 prevalence estimation. Our investigations uncover how increasing vaccination coverage levels, dynamically changing circulating variants, and evolving population immunity levels are important factors in understanding the prevalence-to-wastewater relationship (20).

Exploring in further detail the evolution (over time) of correlations between SARS-CoV-2 prevalence in the population and viral particle concentration in wastewater, and the corresponding ratio of prevalence to wastewater concentration, we found strong correspondence between the survey-based prevalence and the wastewater concentrations during the early studied epidemic periods with low prevalence levels and negligible vaccination of the population. Nevertheless, as mentioned above, we subsequently identified two identifiable factors in a temporally evolving prevalence-to-wastewater relationship: (i) levels of vaccination coverage and (ii) differences in fecal shedding according to predominant variants.

There exists a myriad of potential external influences (or confounders) on the relationship such as changing population-level immunity and human mobility patterns in response to time-varying nonpharmaceutical intervals and the epidemic trajectory (21). However, reduced population-level fecal shedding following widespread vaccination is consistent with findings from community-based wastewater surveillance of COVID-19 in educational facilities (22). On the other hand, variant-specific fecal shedding rates (specifically lower shedding rates during the Omicron period) may have led to the observed modifications to the relationship between wastewater concentrations and prevalence. However, the initial breakdowns in the relationship at the beginning of the Omicron wave cannot be disentangled from the greater background vaccination and immunity, compared with previous waves. Indeed, changes in the relationship had already been observed in the preceding months (from September 2021), possibly due to changes to the dominant SARS-CoV-2 variant (as the Delta variant became dominant in the English population) (18). Nevertheless, the relationship between wastewater concentrations and REACT-1 prevalence estimates is stronger (and more consistent) once again when we restrict measurement focus to the three rounds where Omicron (BA.1 and BA.2) is the dominant SARS-CoV-2 variant, which may be indicative of a returning stabilization of the prevalence-to-wastewater relationship as the population-level fecal shedding settled at reduced levels. Our findings of implied reduced fecal shedding during the Omicron wave were similarly observed in a community-based analysis of wastewater in the United States (23). While our findings are at the population (rather than individual) level, they are also consistent with clinical data for the Omicron variant which indicate preferential infection of the upper airway, selective reduction in Omicron infectivity in the intestinal tract, as well as lower viral loads and shorter duration of respiratory shedding, compared with the Delta variant (14–16, 24).

Leaning now on the results of our modeling analysis, across both periods of our analysis (which are characterized by highly contrasting epidemic dynamics and differing intensities of wastewater surveillance), a trained wastewater-based gradient boosting model with iteratively produced prevalence estimates yielded moderate predictive accuracy for estimating survey-based, high spatial resolution (LTLA-level) prevalence for individual rounds (i.e. up to 1 month) in settings without any concurrent prevalence survey data. Interpreting these findings alongside the stronger predictive accuracy in the coarser spatial resolution of regional level, we deduce that reliably estimating SARS-CoV-2 prevalence from a (recently) calibrated wastewater model alone for a single survey round (i.e. for up to 1 month) is a feasible task.

While wastewater-model-based multistep prevalence estimates (i.e. produced using wastewater data alone for several individual rounds without any recalibrations of the prevalence-to-wastewater relationship) in the *early* period (2020 July 24 to 2021 May 3) generally provided representative accounts of out-of-sample LTLA level and regional trends for up to 3 months (without a simultaneous prevalence survey), there was a notably different picture in the *late* period (2021 May 20 to 2022 March 31). In particular, in the *late* period, characterized by high prevalence levels, increasing vaccination coverage, and changing variants, the multistep estimates failed to provide an accurate account of the substantial prevalence movements present throughout the Omicron wave, at both LTLA and regional spatial resolutions. Such weak out-of-sample predictive performance (when the wastewater model was not recalibrated for ∼3 months) may be a consequence of the increasingly temporally unstable relationship between wastewater and prevalence (apparently influenced by both vaccination and the emergence of the Omicron variant). Our identified lack of temporal consistency is compatible with the findings of Xiao et al. (10), where the wastewater-to-clinical cases ratio also changed substantially during the pandemic. In contrast to multistep estimates, our iteratively produced wastewater-model-based prevalence estimates for the Omicron BA.2 peak in round 19 (2022 March 8 to 2022 March 31) were strongly representative of the observed sharp increases in prevalence at LTLA and regional level. Indeed, a single additional round of model recalibration during the Omicron wave enabled more representative estimates of survey-based prevalence. These examples highlight the important lesson; accuracy and utility of wastewater-model-based prevalence estimates relies heavily on understanding any changes to the circulating variants and relatedly, the prevalence-to-wastewater relationship. We deduce the importance of concurrent prevalence surveys for wastewater-based model calibration, alongside the potential use case of WBE which could be undertaken between rounds of a prevalence survey to improve continuity of prevalence estimates, contingent on availability of vaccination and variant tracking data to inform the updating of the prevalence-to-wastewater relationship.

Limitations of our analysis include the sparse geographical coverage of wastewater collection sites earlier in the pandemic, sensitivity of the wastewater surveillance (and the unknown varying sampling strategies) for quantifying RNA concentrations (25), and imprecision of the REACT-1 prevalence survey data at the LTLA level, as well as possible inaccuracies in the weighted estimates employed to correct for variable response rates in different sectors and demographics of the population (26). Further limitations (discussed in Supplementary Materials 6 and 8) include the temporal resolutions of our data, the discrete time periods of data collection, the lack of adjustment for variability in estimated wastewater concentrations and prevalence estimates, and the spatial and temporal mismatches which result in averaging of concentrations across treatment plants and across a survey round. Additionally, we acknowledge that our analysis of the utility WBE is strictly confined to the use case within a nation where a community prevalence survey (REACT-1) exists to calibrate the prevalence-to-wastewater relationship.

Focusing now on the complementary role of WBE, by leveraging the estimated spatial consistency of the prevalence-to-wastewater relationship (Fig. S6), we show that wastewater-based modeling can improve logistical and economic efficiency of prevalence surveys by providing prevalence estimates over sustained periods for geographies not covered by prevalence surveys. Similarly, relative to spatially smoothed survey-based prevalence, for simulations of reduced survey-round sample size, combined survey-wastewater-based estimates consistently improved upon the accuracy of prevalence levels from reduced surveys alone, even for extremely small-sized surveys. Therefore, we demonstrate that combining WBE with intermittent survey data or fewer samples of survey data may help to maximize the geospatial reach, accuracy, and value of the information, while reducing the cost. Thus, WBE data could be used to provide additional, reliable information to fill in the spatial gaps in estimating SARS-CoV-2 prevalence during intervals with reduced survey data. This could be a cost-effective approach, since the WBE data are relatively cheap to obtain compared with conducting population surveys. Further work including an economic evaluation would be needed to model different scenarios based on timing, extent, and duration of survey data and geospatial coverage of the wastewater data. While this is beyond the scope of the current work, briefly, a cost-benefit analysis for Germany estimated that the national cost for WBE surveillance reagents would be only 0.014% of those required for clinical testing (27), although this did not account for the relatively unknown value (accuracy) of information from WBE compared with prevalence survey data. We recognize that community prevalence studies were not a feature of many nations’ response to the COVID-19 pandemic and thus, it is somewhat unclear how WBE could be used to estimate SARS-CoV-2 prevalence in the absence of prevalence surveys in a nation. While beyond the scope of the current research, we tentatively suggest that in this scenario, WBE could be used for well-documented alternative uses (such as early warning detection and variant tracking (23, 28)) or concurrent prevalence–wastewater analyses from other nations may be used to calibrate how the wastewater concentrations correspond to community prevalence.

From a public health policy perspective, we have uncovered several important lessons which may be useful for future infectious disease outbreaks. These lessons include that using WBE alone may be insufficient for modeling survey-based SARS-CoV-2 prevalence over several months without any concurrent prevalence survey, potentially hampered by limitations (including wastewater uncertainty, survey-based uncertainty, and/or spatiotemporal mismatches). However, for time horizons up to 1 month, contingent on recent survey-based calibration of the prevalence-to-wastewater relationship, a wastewater-based model can fill any temporal gaps in surveillance between survey rounds by providing useful, reliable information on prevalence trends in both fine and coarse spatial resolutions. Indeed, reliable wastewater-model-based prevalence estimation, at fine or coarser spatial resolution is often contingent on recent model calibration (via community prevalence surveys), with the regularity of recalibration determined by factors including changing dominant variants and changing vaccination levels. Importantly, wastewater-based modeling can evidently play a complementary role in improving the cost efficiency of prevalence surveys by also filling gaps in spatial coverage of prevalence surveys or by enhancing accuracy of reduced-size surveys. Thus, there is clear value in the ability of WBE to complement prevalence surveys and achieve greater cost efficiencies. Therefore, we deduce that the appropriateness of WBE in England is dependent on the precise use case (WBE alone or complementary to prevalence surveys), the spatial resolution (coarse or fine), and the concurrent epidemic dynamics (such as vaccination and variants). In the event of future outbreaks of infectious diseases, a combination of surveillance by survey data and WBE is likely to be a cost-effective approach to obtaining situational awareness for policy makers. This will likely require concomitant monitoring of vaccination coverage and variants (accessible via prevalence surveys, WBE, and genotyping), alongside clinical indicators of epidemic dynamics. Together, these will help to determine the optimal timing of wastewater model recalibration and the scale of survey and wastewater monitoring required ensuring representative prevalence estimation.

## Materials and methods

### Study design

The objectives of the study were 2-fold: (i) to establish how SARS-CoV-2 wastewater concentration estimates related to corresponding SARS-CoV-2 community prevalence estimates and (ii) how wastewater concentrations could be effectively used, possibly alongside community prevalence surveys, for monitoring SARS-CoV-2 prevalence in varying spatial resolution across England.

Wastewater concentration data were sourced from the EMHP program, which was led by the United Kingdom Health Security Agency and tested untreated sewage across England for fragments of SARS-CoV-2. The program monitored wastewater viral concentrations of SARS-CoV-2 RNA, variants of concern, and variants under investigation. For estimation of wastewater concentrations, samples were collected three to four times weekly at sewage treatment works (STWs) and sewer network sites (18). The monitoring program commenced in July 2020 at 45 STWs and a large-scale expansion occurred nationally in June 2021. The wastewater samples were obtained in one of two ways: either via (i) grab samples (a single sample taken using a small container at one point in the day) or (ii) composite samples (an auto sampler samples at regular intervals throughout the day and mixes the samples together in a container). The program did not disclose details of the method used (grab or auto sampler) for individual samples. See Supplementary Material 8 for discussion of further sources of wastewater uncertainty and potential inaccuracies (18, 25).

Viral concentrations of SARS-CoV-2 RNA were obtained from wastewater samples by quantifying the number of SARS-CoV-2 N1 gene copies per liter via the RT-qPCR process, a quantification method which combines two main steps of RT and qPCR (6). Normalization of wastewater flows is necessary to account for precipitation dilution of concentrations of SARS-CoV-2 RNA. The indirect normalization conducted under the EMHP program is described elsewhere (25), but briefly, the technique assumed that wastewater flows were not directly observable, flow variability was estimated, and outliers were identified. The data were then presented as the flow-normalized viral concentrations of SARS-CoV-2 RNA, defined in units of numbers of gene copies per liter.

Community prevalence data were sourced from the REACT-1 study which obtained estimates of weighted prevalence of SARS-CoV-2 infection in England from 2020 May 1 to 2022 March 31. Across 19 distinct rounds of cross-sectional surveys (29), random samples of the English population (over 5 years of age) were taken (26, 30). Rounds of REACT-1 were approximately monthly with round durations between 15 and 31 days. The study involved participants using a self-administered throat-and-nose swab kit and completing a questionnaire. The swabs were sent to a laboratory (either by courier or by priority post) where the samples were tested for SARS-CoV-2 by RT-PCR. The laboratory extracted nucleic acid for SARS-CoV-2 using the ViroBOAR 1.0 RT-qPCR kit (EuroFins Genomics, GmbH, Ebersberg, Germany) and the Roche Lightcycler 480 II (Roche Diagnostics, Almere, The Netherlands) to detect in parallel two gene targets: the N gene and E gene. Valid laboratory test results were defined as positive for an individual if both the N gene and E gene targets were detected or if the N gene was detected with a cycle threshold (Ct) value <37. Date of swabbing was used for prevalence estimates where available, and otherwise, the study used date of pickup by courier or date of scan in post office (26, 30).

Throughout the 19 rounds, there were ∼2.5 million respondents who had a valid laboratory RT-PCR test for SARS-CoV-2. The use of random sampling of individuals in the study was advantageous as it avoided many of the inherent biases that exist with only testing of symptomatic individuals, test-seeking behaviors, and the availability of tests. Nevertheless, the REACT-1 study also used a within-round random iterative method, weighting to correct prevalence estimates to be representative of the population (and thus, account for possible nonresponse bias) (31). The weighting was adjusted for gender, age, LTLA counts, deprivation status, and ethnic group (30). We provide more extensive details of the REACT-1 study design, sampling strategy, and statistical analyses in Supplementary Material 7.

### Geospatial mapping of wastewater concentrations and relating to REACT-1

In our analysis, our temporal resolution is individual REACT-1 survey rounds. For each survey round, we took the median value of an STW's flow-normalized viral concentrations (henceforth termed the *concentrations*), which are then log-transformed. In terms of spatial resolution, the catchment areas of STW locations for the reported concentrations do not align with the spatial resolution of the REACT-1 study; the 315 LTLAs of England. We therefore used a geospatial approach to align the spatial resolutions, i.e. to map the wastewater concentrations from the STW level to LTLA level (of the prevalence survey). We employed the lookup table provided by Hoffmann et al. (32), which gives a mapping of wastewater catchment areas to lower layer super output areas (LSOAs); small regional geographies that combine to form the larger geography of an LTLA. For 21 STWs of the EMHP program which are not included in the lookup table, to estimate their catchment areas, we employed an approximation method proposed by Li et al. (12).

Equipped with the catchment-to-LSOA mapping area data and Office for National Statistics 2019 mid-year population estimates, we then derived geospatial population estimates (GPEs) for the intersection area between each catchment area of STW i and LTLA k. Our geospatial approach is adapted from Hoffmann et al. (32), and we assumed that each LSOA population is uniformly distributed across the area serviced by STWs:


$$\hat{P_{k}}=\sum_{j=1}^{J}\hat{p_{j}},$$

where $\hat{P_{k}}$ is the GPE for LTLA k and $\hat{p_{j}}$ is our estimate of the serviced population of LSOA j based on our population estimates for the intersection area between the catchment area of STW i and LSOA j (see Supplementary Material 6 for further details).

For each LTLA k, the weight $w_{ik}$ assigned to STW i for concentrations was the ratio of the GPE for the intersection area between the catchment area of STW i and area of LTLA k divided by the GPE for LTLA k:


$$w_{ik}=\frac{\sum_{j=1}^{J}\hat{g_{ij}}}{\hat{P_{k}}},$$

where $\hat{g_{ij}}$ is the GPEs for the intersection area between the catchment area of STW i and area of LSOA j. If any particular STW s failed to report concentrations within a round of the REACT-1 study, the weights of LTLA k for that round were re-weighted such that $\sum_{i\neq s}w_{ik}=1\;\mathrm{and}\;w_{sk}=0$.

Finally, for a given round, the estimated concentration of an LTLA was the weighted average of the median STW-level concentrations within that LTLA. The weights employed here were the STW–LTLA population estimates. We also (Supplementary Material 9) considered alternative approaches such as taking the wastewater measurement of the (single) dominant STW in an LTLA, the mean STW-level concentration across the round (as opposed to the median), and various lead/lag times where we expanded/reduced the window for wastewater measurements to account for potential early or prolonged wastewater-based detection of SARS-CoV-2. Each alternative approach did not led to an enhanced relationship between the estimated wastewater concentrations and REACT-1 prevalence estimates.

In the event of future outbreaks of infectious diseases, the developed approach could be applicable to population-level wastewater analyses which require spatial alignment of wastewater catchments to geographies used by public health authorities. Nevertheless, there exists limitations with the predictive approach of using GPEs, such as transient populations, uncertainty surrounding population estimates of small geographic areas, and potentially unrepresentative wastewater concentration estimates for some LTLAs, each of which are discussed in Supplementary Material 6.

### Relationship between wastewater concentrations and REACT-1 prevalence

Our spatiotemporal analyses are divided into two distinct periods; the *early* period of rounds 3 to 11 (2020 July 24 to 2021 May 3—the first time periods where the EMHP program intersects with the REACT-1 study) and the *late* period of rounds 12 to 19 (2021 May 20 to 2022 March 31). The two periods are primarily analyzed independently due to differences in wastewater surveillance geographic coverage and the EMHP program's transition from usage of two laboratories to a single laboratory.

To allow for any potential nonlinearities, Spearman's correlation was used to assess the relationship between (log) concentration and SARS-CoV-2 prevalence. Note that log concentration, as opposed to concentration, was used in these analyses as this variable served as our predictor within our best-performing wastewater-based gradient boosting models (see below). In particular, we studied correlations between all LTLA-level (and regional population-weighted averages of) SARS-CoV-2 wastewater concentration estimates and REACT-1 SARS-CoV-2 prevalence estimates (i) across the *early* and *late* periods separately and (ii) across moving five-round windows centered on each survey round from REACT-1 rounds 5 (starting 2020 September 18) to 17 (ending 2022 January 20). Then, for comprehensiveness, we also assessed the correlations within the time series for each individual LTLA (and each region) to understand any variability in the relationships for different geographies nationally.

### Modeling REACT-1 SARS-CoV-2 prevalence

Our primary model for estimating LTLA-level SARS-CoV-2 prevalence is an extreme gradient boosting model which is implemented via the xgboost package in R version 4.2.1 (33, 34). Briefly, the extreme gradient boosting (known as XGBoost) machine learning algorithm is comprised of an ensemble of weak learners consecutively fit to data in a greedy manner. The algorithm is a parallelized, optimized version of the general gradient boosting algorithm (35). XGBoost was chosen due to its predictive capabilities and highly flexible nature (ability to handle complex nonlinearities and time-varying relationships such as the prevalence-to-wastewater relationship over the course of the COVID-19 pandemic), while also enabling regularization (via model hyperparameters such as the learning rate) and cross-validation. Indeed, XGBoost often produces state-of-the-art predictive performance across a variety of settings, including for our research objective of estimating SARS-CoV-2 prevalence from wastewater concentrations (11). We developed a Bayesian hierarchical model to ensure robustness of conclusions from our modeling analysis (described in Supplementary Material 10). Vaccination data from REACT-1 were used as covariates in the *late* period, and a full description of covariates used in our wastewater-based models of SARS-CoV-2 prevalence is provided in Table S2. 95% prediction intervals (PIs) were obtained using 5,000 nonparametric bootstrap samples/replicates of the original training sets.

### Types of wastewater-model-based estimates

Our appraisal of model performance focused on wastewater-model-based estimates of SARS-CoV-2 prevalence. Note that we only trained our model at an LTLA spatial resolution (as model training at a regional level produced inferior accuracy in regional estimates to those obtained by training at an LTLA level). Regional estimates were achieved by weighting the LTLA-level estimates by the corresponding LTLA populations within the region.

There were three broad types of scenarios for wastewater-model-based prevalence estimates: (i) *Iterative training and testing* involved calibrating our wastewater-based model using a minimum of four REACT-1 rounds and subsequently using wastewater data in our model to estimate prevalence for a full REACT-1 round (for which we omitted prevalence data) one-at-a-time. In further detail, we made use of wastewater and prevalence data up to one survey round before each testing round, and then our wastewater-based model used the testing round wastewater data to produce prevalence estimates in each testing round. The idea was to evaluate inferences gained from subsequently relying on WBE alone (for up to 1 month) or temporarily between rounds of a prevalence survey. (ii) *Multistep testing* involved training our wastewater model for five to six survey rounds but using wastewater data to estimate prevalence for three individual survey rounds (for which prevalence data was omitted for testing) without recalibrating the model. In other words, the model learned the relationship between wastewater and prevalence during training rounds, and then used wastewater data alone (within the model) during model testing for estimation of prevalence within the three individual survey rounds. The intuition here was to appraise the exclusive application of WBE over an ∼3-month period without any concurrent REACT-1 survey data to recalibrate the prevalence-to-wastewater relationship. (iii) *Complementary wastewater-based estimation* differed to the cases above as it did not omit all of the testing rounds’ prevalence data and hence, involved partially (counterfactually) complete, survey rounds. In doing so, we assessed the effectiveness of WBE for complementing incomplete prevalence surveys which could arise due to funding and/or logistical limitations which prevent a large-scale, national survey program. Here, we (ⅰ) first considered reduced geographic coverage of prevalence surveys as we excluded varying numbers of random LTLAs from single survey rounds, and the corresponding prevalence levels were estimated using our wastewater-based model and compared with corresponding REACT-1 prevalence estimates. (ⅱ) Second, we investigated how WBE could contribute to prevalence estimation in the event of reduced survey sample size totals (i.e. fewer survey participants) within rounds. Using reduced survey-round totals of participants, we simulated the number of positive individuals in an LTLA (within a round) from a binomial distribution with success probability equal to the REACT-1 weighted prevalence. We replicated the simulation 100 times to reduce sensitivity to sources of randomness. A key challenge in the simulation analysis was to decide an appropriate benchmark against which we could compare the accuracy of prevalence estimates, in survey rounds of varying sizes, with and without a wastewater model. The lack of an external benchmark to validate estimates is a key limitation of our simulated reduced sample size analysis. In the absence of knowing the true prevalence in communities (the ideal benchmark), we used a pseudo-independent, spatially smoothed version of reported REACT-1 prevalence levels (i.e. spatially smoothing the full-sized survey-based prevalence estimates). In doing so, we attempted to reduce the likelihood of bias and misleading conclusions potentially caused by benchmarking reduced-survey estimates against either complete survey-based prevalence estimates or completely wastewater-model-based estimates, while further considering the potential presence of spatial autocorrelation in SARS-CoV-2 prevalence. Briefly, the degree of spatial smoothing of LTLA-level prevalence observations within each round was determined by fitting of simple Kriging models to each of the 309 observations and the distances between LTLA centroids, thus accounting for the degree of spatial autocorrelation in prevalence levels within each survey round. Sensitivity of inferences to the choice of spatial smoothing method was appraised by also smoothing according to a smoothing kernel.

Due to our study's objective of assessing the utility of WBE for complementing or replacing survey-based prevalence estimates, the predictive accuracy of wastewater-model-based estimates (i.e. performance of our model on new, unseen data) was appraised relative to REACT-1 SARS-CoV-2 prevalence estimates (unsmoothed unless stated otherwise), using metrics such as MAE, Pearson's correlation with the response (*r*), and accuracy in detecting directional prevalence movements. As MAE is generally dependent on the prevalence levels of a specific round, we often report the mean prevalence level to provide context for each attained MAE. Note that we also assessed LTLA-specific predictive accuracy by focusing on the estimates for each LTLA/region individually (i.e. stratifying the time series) and then reporting summary statistics across the LTLA/region-specific performance metrics. The intuition for assessing LTLA/region-specific predictive accuracy is that we may be interested in tracking the prevalence trends of individual geographies over time.

## Supplementary Material

pgae438_Supplementary_Data

## Data Availability

Access to REACT-1 individual-level data is restricted to protect participants’ anonymity. We obtained LTLA-level REACT-1 data under data request. The wastewater concentration surveillance data from the EMHP are publicly available at https://www.gov.uk. The wastewater catchment areas for the United Kingdom are available at https://github.com/tillahoffmann/wastewater-catchment-areas.

## Preprints

This manuscript was originally published as a preprint at https://doi.org/10.1101/2023.08.17.23293589.
