# Supplementary material for: The utility of wastewater surveillance for monitoring SARS-CoV-2 prevalence
Source: PNAS Nexus. 2024 Oct 4;3(10):pgae438. doi: 10.1093/pnasnexus/pgae438 (PMC11518864; doi:10.1093/pnasnexus/pgae438)
Supplement: pgae438_Supplementary_Data [file pgae438_supplementary_data.pdf]

Supplementary Material 1 Relationship Between SARS-CoV-2 RNA Wastewater Concentrations and Prevalence

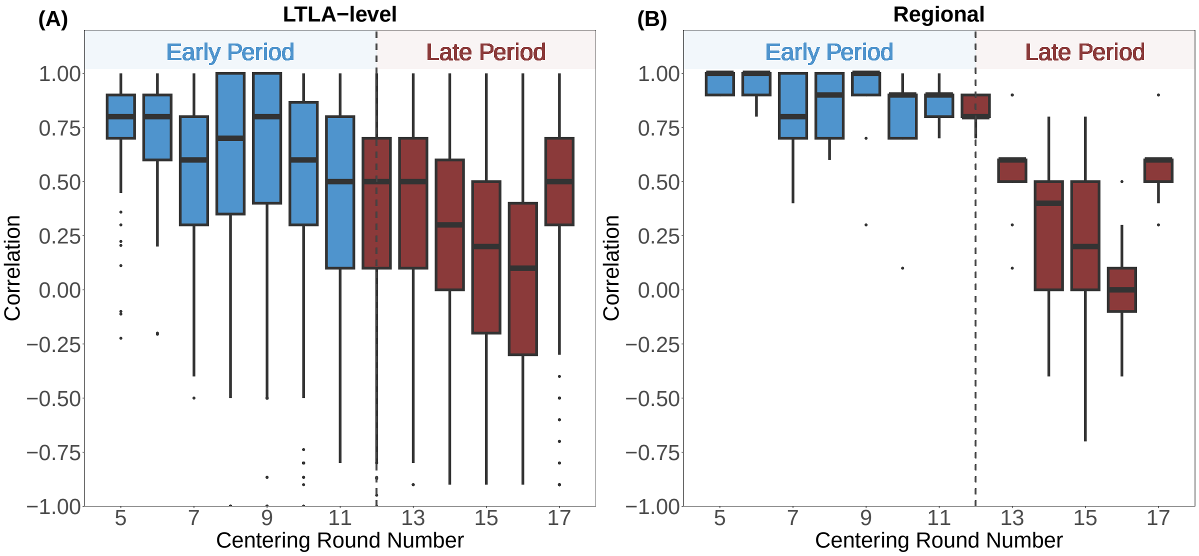

Figure SI 1: **Prevalence-wastewater relationship within LTLAs and Regions.** This figure is similar to Figure 1 B-C, but here we compute Spearman's correlation between SARS-CoV-2 wastewater concentrations and REACT-1 SARS-CoV-2 prevalence for each LTLA and each region separately. Again, correlations were calculated within moving five-round windows centred on each survey round from REACT-1 rounds 5 (18 September 2020) to 17 (5 January 2022). Then, we present the results in boxplot visualisations, where the upper and lower hinges represent the first and third quartiles and the horizontal solid black line represents the median correlation. Whilst there are $n = 9$ regional correlations for each 5-round window, the number of LTLA-level correlations varies by survey round depending on the number of LTLAs included in the wastewater surveillance programme.

Table SI 1: **Correlation analysis, by period, for wastewater concentrations and prevalence.** Spearman's correlation (r) is measured across all observations for each study period, where n is the number of observations used to calculate the corresponding correlation at each spatial resolution and a 95% CI is approximated using a paired bootstrap of 1000 replicates. Among LTLAs which report at least three wastewater concentration measurements across the early period - rounds 3 to 11 (24 July 2020 to 3 May 2021), the median LTLA-level correlation is 0.72, calculated as the median of each LTLA's individual correlation between wastewater concentrations and prevalence levels across the rounds. The corresponding median of the LTLA-specific correlations between concentration-vaccination interaction and SARS-CoV-2 prevalence is 0.79 across the late period - rounds 12 to 19 (20 May 2021 to 31 March 2022). In further detail, these medians of the LTLA-specific correlations can be interpreted as the average of LTLA-specific relationships between wastewater concentrations and prevalence. The regional and national-level SARS-CoV-2 wastewater concentration (and concentration-vaccination interaction) and REACT-1 prevalence estimates were obtained by population-level weighting. Here, for each period, we measured the correlation between the observed values of such regional and national average concentrations and corresponding prevalence. Note that we defined here (and in our modelling analysis) the concentration-vaccination interaction using the proportion of survey participants that were fully vaccinated (two or more doses) from the previous survey round, and the concentration from the current survey round (due to the apparent time taken for vaccination to impact on subsequent population-level faecal shedding).

**Rounds**	**Variable**	**Spatial Resolution**	**n**	**r (95% CI)**
3-11	Concentration	LTLA	1572	0.62 (0.59, 0.65)
3-11	Concentration	Regional	81	0.83 (0.75, 0.89)
3-11	Concentration	National	9	0.98 (0.82, 1.00)
12-19	Concentration-Vaccination Interaction	LTLA	2461	0.71 (0.69, 0.73)
12-19	Concentration-Vaccination Interaction	Regional	72	0.89 (0.79, 0.94)
12-19	Concentration-Vaccination Interaction	National	8	0.93 (0.60, 1.00)

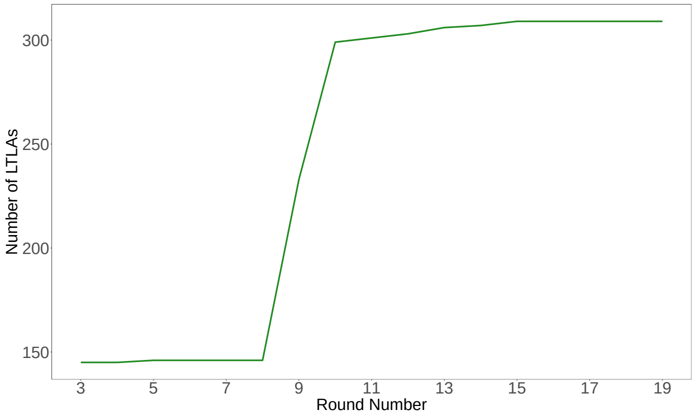

Figure SI 2: **Number of studied LTLAs by survey round of REACT-1.** The number of LTLA-level observations varied by survey round depending on the number of LTLAs included in the wastewater surveillance programme. Here, we visualise the number of such LTLAs across REACT-1 rounds 3 to 19 (24 July 2020 to 31 March 2022).

Supplementary Material 2 Wastewater-Based Model Covariates

Table SI 2: **Covariates used in wastewater-based models for estimating SARS-CoV-2 prevalence**. Unless otherwise stated, all covariates are at an LTLA level. Data originate from the REACT-1 study and the EMHP surveillance programme, aside from the LTLA population estimates (provided by the ONS) which are used as weights for regional and national predictors.

Variable	Units	Description
Wastewater Concentration	Log Gene copies per litre (gc/l)	Estimated LTLA-level wastewater viral concentrations per round. Concentrations are obtained via the weighted contribution of each treatment plant's average (normalised) concentration for the round (described in Materials and Methods).
Neighbour-Averaged Concentration	Log Gene copies per litre (gc/l)	A spatial weights matrix (with row standardisation) is computed based on the neighbourhood structure of LTLAs, using queen contiguity criterion. The weights of this sparse matrix are multiplied by the LTLAs' concentrations for each round to yield a weighted average of the neighbouring areas' concentrations.
Vaccination-Log Concentration Interaction	Log Gene copies per litre (gc/l)	(Fully Vaccinated Proportion) × (Estimated Concentration). Fully vaccinated proportions are the proportion of the LTLA population estimated (by the REACT-1 study) to have received two or more vaccination doses, whilst concentrations are the estimated LTLA-level concentration.
Regional Average Concentration	Log Gene copies per litre (gc/l)	Average of a region's wastewater concentrations for a round, weighted by the underlying LTLA populations (for whom measurements were available in that round).
National Average Concentration	Log Gene copies per litre (gc/l)	National average of wastewater concentrations for a round, weighted by the underlying LTLA populations (for whom measurements were available in that round).
Concentration Difference	Log Gene copies per litre (gc/l)	The change in an LTLA's wastewater concentration from one round to the next.
National Concentration Difference	Log Gene copies per litre (gc/l)	The change in the national average wastewater concentration from one round to the next.
Historical Regional Prevalence per Log Concentration	% per log gc/l	For each LTLA, prevalence per log concentration is calculated, and the corresponding regional average is the weighted average of the LTLA-level values for that round (where the weights are the LTLA populations). The regional average across the training period is used as the predictor for the testing set. Region-level prevalence per log concentration is used due to the noisiness of the LTLA-level estimates.

Supplementary Material 3 Out-of-Sample Wastewater-Model-Based Estimates of Prevalence

The following section presents the tables and figures which complement the results (see main text) of using a wastewater-based model to estimate SARS-CoV-2 for individual survey rounds which are out-of-sample in the sense that the prevalence data is excluded for all LTLAs in that testing round (and all subsequent survey rounds).

Table SI 3: **Within-round predictive performance of iteratively-updating wastewater-based model for REACT-1 rounds 7-11 (13 November 2020 to 3 May 2021)**. Summary of accuracy metrics for wastewater-based model's out-of-sample prevalence estimates, where the model was trained iteratively in the sense that the model training window expands over time, to add one additional training round, for each individual testing round. Estimates were out-of-sample in the sense that the prevalence data was omitted for the individual testing round (and all subsequent survey rounds). The intuition is to assess how well a wastewater-based model could estimate SARS-CoV-2 prevalence for a relatively short period of time, such as temporarily between rounds of a prevalence survey. The wastewater-based (gradient boosting) model was trained at an LTLA level, and regional-level estimates were obtained by weighting the LTLA-level estimates by their corresponding populations within the regions. Here, accuracy metrics have the interpretation that they measure the within-round predictive performance across all n observations, where n is either the number of LTLAs covered by the wastewater surveillance programme or is equal to nine (the number of regions). *Top 25 Common* is the number of LTLAs common to the highest predicted prevalence levels and REACT-1 prevalence levels. *Change Detection* indicates the mean directional accuracy, whilst the corresponding 95% CI is attained by Clopper-Pearson method, otherwise known as the Exact Confidence Interval (1). *MAE* represents the mean absolute error between each round's model-based predictions and the corresponding LTLA-level (or regional population-weighted average) REACT-1 prevalence estimates. *Mean Prevalence* is the mean average of the individual rounds' LTLA-level (regional population-weighted) REACT-1 prevalence estimates, and is cited as a guide for appraisal of MAE within rounds. Results at a regional level are visualised in Figure SI 3.

Testing Round	Training Rounds	Resolution	n	Top 25 Common	Change Detection (95% CI)	MAE	Mean Prevalence
7	3-6	LTLA	146	4	71.9% (63.9%,79.0%)	0.57%	1.00%
8	3-7	LTLA	146	3	72.6% (64.6%,79.7%)	0.9%	1.82%
9	3-8	LTLA	233	8	83.7% (78.3%,88.2%)	0.41%	0.49%
10	3-9	LTLA	299	8	72.6% (67.1%,77.6%)	0.23%	0.19%
11	3-10	LTLA	301	3	59.8% (54.0%,65.4%)	0.13%	0.11%
7	3-6	Regional	9		77.8% (40.0%,97.2%)	0.28%	0.97%
8	3-7	Regional	9		88.9% (51.8%,99.7%)	0.56%	1.50%
9	3-8	Regional	9		88.9% (51.8%,99.7%)	0.31%	0.52%
10	3-9	Regional	9		100.0% (66.4%,100.0%)	0.13%	0.22%
11	3-10	Regional	9		88.9% (51.8%,99.7%)	0.08%	0.10%

Table SI 4: **Predictive performance of iteratively-updating wastewater-based model over time for REACT-1 rounds 7-11 (13 November 2020 to 3 May 2021)**. Summary of accuracy metrics, over five rounds, for a wastewater-based model, which was trained iteratively - in the sense that the model training window expands over time, to add one additional training round, for each individual testing round. Estimates were out-of-sample in the sense that the prevalence data was omitted for each individual testing round (and all subsequent survey rounds). The intuition was to assess how well a wastewater-based model could estimate SARS-CoV-2 prevalence for a relatively short period of time, such as temporarily between rounds of a prevalence survey. The wastewater-based (gradient boosting) model was trained at an LTLA level, and regional-level estimates were obtained by weighting the LTLA-level estimates by their corresponding populations. Here, accuracy metrics have the interpretation that they measure the predictive performance over time (i.e. five rounds) across all $n^{(obs)}$ observations. *Change Detection* indicates the mean directional accuracy, whilst the corresponding 95% CI is attained by Clopper-Pearson method, otherwise known as the Exact Confidence Interval (1). r denotes the Pearson's correlation between all wastewater-model-based estimates and REACT-1 SARS-CoV-2 prevalence. *MAE* represents the mean absolute error between all model-based predictions and the corresponding LTLA-level (or regional population-weighted average) REACT-1 prevalence estimates. *Mean Prevalence* is the mean average of the LTLA-level (regional population-weighted) REACT-1 prevalence estimates, and is cited as a guide for appraisal of MAE across the rounds. $n^{(geo)}$ denotes the number of geographies covered by both the wastewater surveillance and community prevalence surveys. Median r (Q1, Q3) denotes the median of the LTLA-level/regional Pearson's correlations between their wastewater-model-based estimates and their corresponding REACT-1 SARS-CoV-2 prevalence. This summary statistic may be useful for understanding how well our wastewater model could track the prevalence trends of individual LTLAs (or regions) for these short periods of time of up to month (and therefore, potentially in between rounds of a community prevalence survey). Results at a regional level are visualised in Figure SI 3.

Testing Rounds	Training Rounds	Resolution	$n^{(obs)}$	Change Detection (95% CI)	r	MAE	Mean Prevalence	$n^{(geo)}$	Median r (Q1,Q3)
7-11	3-10	LTLA	1125	71.4% (68.6%,74.0%)	0.66	0.37%	0.54%	299	0.85 (0.62, 0.96)
7-11	3-10	Regional	45	88.9% (75.9%,96.3%)	0.79	0.27%	0.66%	9	0.94 (0.85, 0.95)

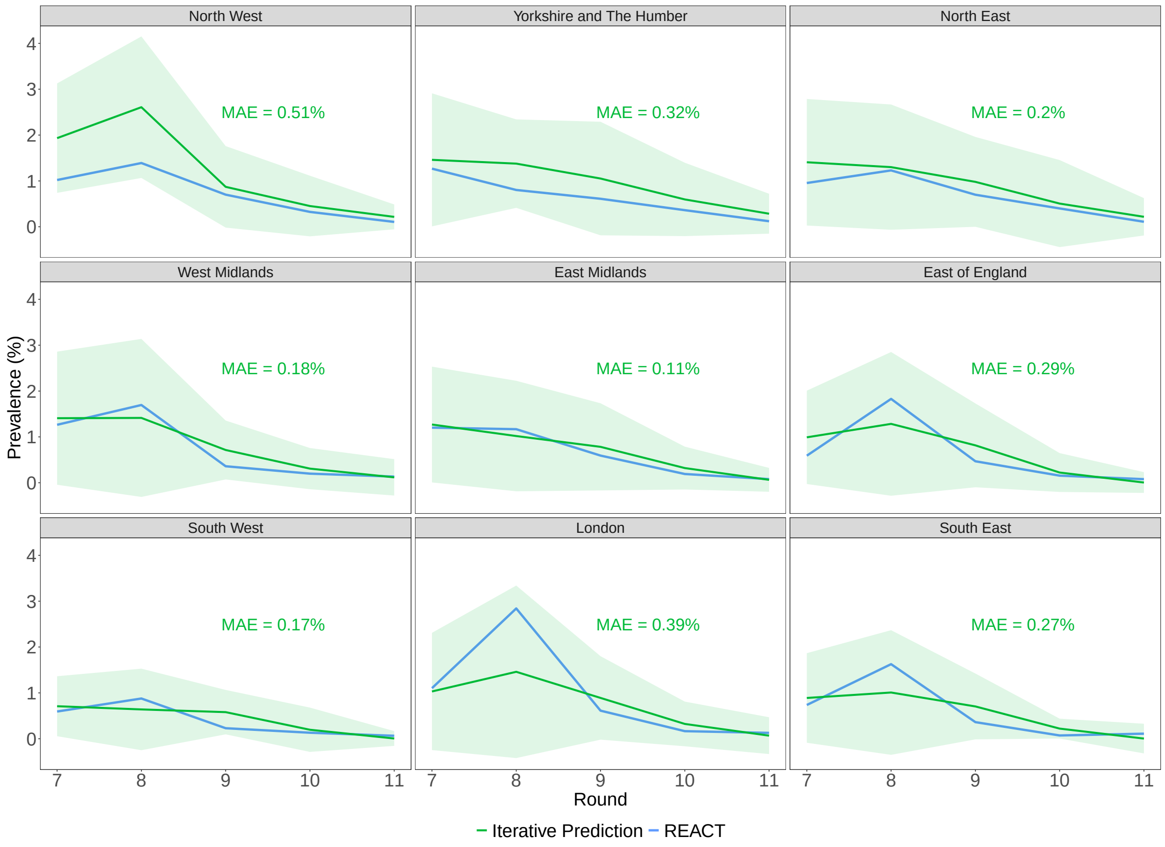

Figure SI 3: **Regional out-of-sample, wastewater-model-based prevalence estimates in rounds 7 to 11 (13 November 2020 to 3 May 2021).** Wastewater-model-based prevalence estimates are shown alongside REACT-1 prevalence (blue). Estimates were out-of-sample in the sense that the prevalence data was omitted for each individual testing round (and all subsequent survey rounds). The model was trained at an LTLA level with iterative (green) updating of the model (thus expanding the training set every round with an additional training round). The scenario amounts to employing a wastewater-based model for a single survey round (i.e. a relatively short period of time up to one month), and thus assesses predictive performance with regular model calibration of the wastewater-to-prevalence relationship. Such a scenario may arise in between rounds of a community prevalence survey and thus, captures the ability of wastewater-based models for filling temporal gaps in prevalence surveillance. The wastewater-model-based and REACT-1 prevalence estimates for each region (per individual round) were obtained by weighting the LTLA-level prevalence estimates by the corresponding LTLA populations within the region. The shaded 95% prediction intervals (PIs) were obtained by 5,000 non-parametric bootstrap samples. The mean average of the (population-weighted) regional prevalence values across rounds 7 to 11 was 0.66%.

Table SI 5: **Comparing predictive performance, over time, of an iteratively-updating wastewater-based model's estimates and a wastewater-based model making multi-step prevalence estimates for REACT-1 rounds 9-11 (4 February 2021 to 3 May 2021)**. The wastewater-based model which was trained iteratively meant that the model training window expands over time, to add one additional training round, for each individual testing round. The intuition is to assess how well a wastewater-based model could estimate SARS-CoV-2 prevalence for a relatively short period of time, such as temporarily between rounds of a prevalence survey. On the other hand, the wastewater-based model which made multi-step prevalence estimates had a fixed training set (of rounds 3 to 7) across the three testing rounds and wastewater-model-based prevalence estimates were made for the individual testing rounds (without any updating of the training set). In each case, the wastewater-model-based estimates for each individual round were out-of-sample in the sense that prevalence data was omitted for that individual round (and all subsequent rounds). The wastewater-based (gradient boosting) models were trained at an LTLA level, and regional-level estimates were obtained by weighting the LTLA-level estimates by their corresponding populations. Here, accuracy metrics have the interpretation that they measure the predictive performance over time (i.e. three rounds) across all $n^{(obs)}$ observations. *Change Detection* indicates the mean directional accuracy, whilst the corresponding 95% CI is attained by Clopper-Pearson method, otherwise known as the Exact Confidence Interval (1). r denotes the Pearson's correlation between all wastewater-model-based estimates and REACT-1 SARS-CoV-2 prevalence. *MAE* represents the mean absolute error between all model-based predictions and the corresponding LTLA-level (or regional population-weighted average) REACT-1 prevalence estimates. *Mean Prevalence* is the mean average of the LTLA-level (regional population-weighted) REACT-1 prevalence estimates, and is cited as a guide for appraisal of MAE across the rounds. $n^{(geo)}$ denotes the number of geographies covered by both the wastewater surveillance and community prevalence surveys. Median r (Q1, Q3) denotes the median of the LTLA-level/regional Pearson's correlations between their wastewater-model-based estimates and their corresponding REACT-1 SARS-CoV-2 prevalence. This summary statistic may be useful for understanding how well our wastewater model could track the prevalence trends of individual LTLAs (or regions) for either short periods of time of up to month (and therefore, potentially in between rounds of a community prevalence survey) or possibly, longer periods of up to three months. Results for both modelling environments are visualised at a regional level are visualised in Figure 2.

Type	Test Rounds	Training Rounds	Resolution	$n^{(obs)}$	Change Detection (95% CI)	r	MAE	Mean Prevalence	$n^{(geo)}$	Median r (Q1,Q3)
Iterative	9, 10, 11	3-8, 3-9, 3-10	LTLA	1125	71.1% (67.9%,74.1%)	0.54	0.25%	0.24%	299	0.91 (0.41, 0.99)
Multi-Step	9, 10, 11	3-8	LTLA	1125	69.0% (65.8%,72.2%)	0.52	0.27%	0.24%	299	0.87 (0.38, 0.98)
Iterative	9, 10, 11	3-8, 3-9, 3-10	Regional	27	92.6% (75.7%,99.1%)	0.94	0.17%	0.28%	9	1 (0.99, 1)
Multi-Step	9, 10, 11	3-8	Regional	27	77.8% (57.7%,91.4%)	0.93	0.21%	0.28%	9	0.98 (0.96, 0.99)

Table SI 6: **Within-round predictive performance of iteratively-updating wastewater-based model for REACT-1 rounds 15-19 (19 October 2021 to 31 March 2022)**. Summary of accuracy metrics for wastewater-based model's out-of-sample prevalence estimates, where the model was trained iteratively in the sense that the model training window expands over time, to add one additional training round, for each individual testing round. Estimates were out-of-sample in the sense that the prevalence data was omitted for the individual testing round (and all subsequent survey rounds). The intuition was to assess how well a wastewater-based model could estimate SARS-CoV-2 prevalence for a relatively short period of time, such as temporarily between rounds of a prevalence survey. The wastewater-based (gradient boosting) model was trained at an LTLA level, and regional-level estimates were obtained by weighting the LTLA-level estimates by their corresponding populations within the regions. Here, accuracy metrics have the interpretation that they measure the within-round predictive performance across all n observations, where n is either the number of LTLAs covered by the wastewater surveillance programme or is equal to nine (the number of regions). *Top 25 Common* is the number of LTLAs common to the highest predicted prevalence levels and REACT-1 prevalence levels. *Change Detection* indicates the mean directional accuracy, whilst the corresponding 95% CI is attained by Clopper-Pearson method, otherwise known as the Exact Confidence Interval (1). *MAE* represents the mean absolute error between each round's model-based predictions and the corresponding LTLA-level (or regional population-weighted average) REACT-1 prevalence estimates. *Mean Prevalence* is the mean average of the individual rounds' LTLA-level (regional population-weighted) REACT-1 prevalence estimates, and is cited as a guide for appraisal of MAE within rounds. Results at a regional level are visualised in Figure SI 4.

Testing Round	Training Rounds	Resolution	n	Top 25 Common	Change Detection (95% CI)	MAE	Mean Prevalence
15	3-14	LTLA	309	1	75.1% (69.9%,79.8%)	0.83%	1.58%
16	3-15	LTLA	309	1	74.1% (68.8%,78.9%)	0.79%	1.37%
17	3-16	LTLA	309	2	62.8% (57.1%,68.2%)	2.76%	4.18%
18	3-17	LTLA	309	8	73.8% (68.5%,78.6%)	1.58%	2.84%
19	3-18	LTLA	309	1	91.3% (87.5%,94.2%)	2.31%	6.47%
15	3-14	Regional	9		66.7% (29.9%,92.5%)	0.58%	1.59%
16	3-15	Regional	9		66.7% (29.9%,92.5%)	0.33%	1.37%
17	3-16	Regional	9		66.7% (29.9%,92.5%)	3.17%	4.66%
18	3-17	Regional	9		77.8% (40.0%,97.2%)	1.44%	2.79%
19	3-18	Regional	9		100.0% (66.4%,100.0%)	1.87%	6.26%

Table SI 7: **Predictive performance of iteratively-updating wastewater-based model over time for REACT-1 rounds 15-19 (19 October 2021 to 31 March 2022)**. Summary of accuracy metrics for wastewater-based model, which was trained iteratively - in the sense that the model training window expands over time, to add one additional training round, for each individual testing round. The intuition was to assess how well a wastewater-based model could estimate SARS-CoV-2 prevalence for a relatively short period of time, such as temporarily between rounds of a prevalence survey. Estimates were out-of-sample in the sense that the prevalence data was omitted for the individual testing round (and all subsequent survey rounds). The wastewater-based (gradient boosting) model was trained at an LTLA level, and regional-level estimates were obtained by weighting the LTLA-level estimates by their corresponding populations. Here, accuracy metrics have the interpretation that they measure the predictive performance over time (i.e. five rounds) across all $n^{(\text{obs})}$ observations. *Change Detection* indicates the mean directional accuracy, whilst the corresponding 95% CI is attained by Clopper-Pearson method, otherwise known as the Exact Confidence Interval (1). r denotes the Pearson's correlation between all wastewater-model-based estimates and REACT-1 SARS-CoV-2 prevalence. *MAE* represents the mean absolute error between all model-based predictions and the corresponding LTLA-level (or regional population-weighted average) REACT-1 prevalence estimates. *Mean Prevalence* is the mean average of the LTLA-level (regional population-weighted) REACT-1 prevalence estimates, and is cited as a guide for appraisal of MAE across the rounds. $n^{(\text{geo})}$ denotes the number of geographies covered by both the wastewater surveillance and community prevalence surveys. Median r (Q1, Q3) denotes the median of the LTLA-level/regional Pearson's correlations between their wastewater-model-based estimates and their corresponding REACT-1 SARS-CoV-2 prevalence. This summary statistic may be useful for understanding how well our wastewater model could track the prevalence trends of individual LTLAs (or regions) for these short periods of time of up to month (and therefore, potentially in between rounds of a community prevalence survey). Results at a regional level are visualised in Figure SI 4.

Testing Rounds	Training Rounds	Resolution	$n^{(\text{obs})}$	Change Detection (95% CI)	r	MAE	Mean Prevalence	$n^{(\text{geo})}$	Median r (Q1,Q3)
15, 16, 17, 18, 19	3-14, 3-15, 3-16, 3-17, 3-18	LTLA	1545	75.4% (73.2%,77.5%)	0.65	1.65%	3.29%	309.00	0.78 (0.57, 0.90)
15, 16, 17, 18, 19	3-14, 3-15, 3-16, 3-17, 3-18	Regional	45	75.6% (60.5%,87.1%)	0.74	1.48%	3.33%	9.00	0.75 (0.65, 0.88)

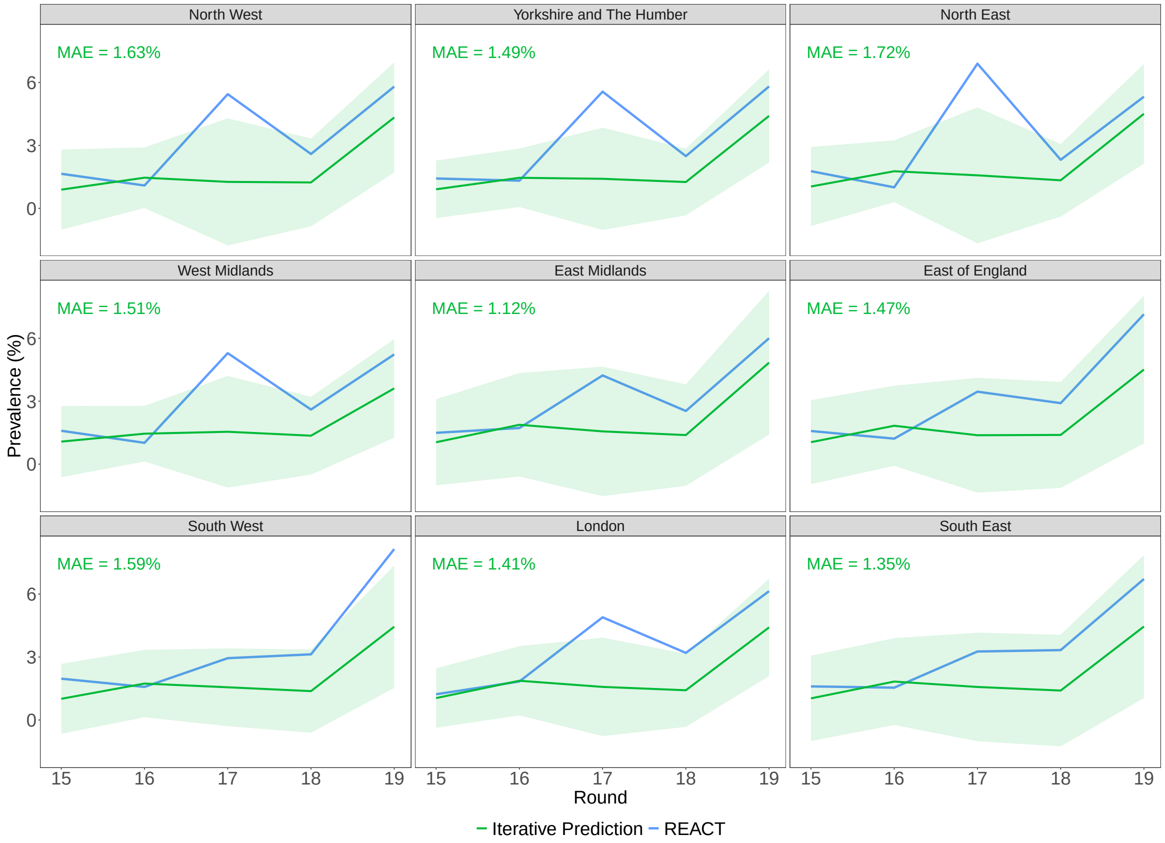

Figure SI 4: **Regional out-of-sample, wastewater-model-based prevalence estimates in rounds 17 to 19 (19 October 2021 to 31 March 2022).** Wastewater-model-based prevalence estimates are shown alongside REACT-1 prevalence (blue). The model was trained at an LTLA level with iterative (green) updating of the model (thus expanding the training set every round with an additional training round). The scenario amounts to employing a wastewater-based model for a single survey round (i.e. a relatively short period of time up to one month), and thus assesses predictive performance with regular model calibration of the wastewater-to-prevalence relationship. Such a scenario may arise in between rounds of a community prevalence survey and thus, captures the ability of wastewater-based models for filling temporal gaps in prevalence surveillance. The wastewater-model-based and REACT-1 prevalence estimates for each region (per individual round) were obtained by weighting the LTLA-level prevalence estimates by the corresponding LTLA populations within the region. The shaded 95% prediction intervals (PIs) were obtained by 5,000 non-parametric bootstrap samples. The mean average of the (population-weighted) regional prevalence values across rounds 15 to 19 was 3.33%.

Table SI 8: **Comparing predictive performance, over time, of iteratively-updating wastewater-based model and wastewater-based model making multi-step prevalence estimates for REACT-1 rounds 17 to 19 (19 October 2021 to 31 March 2022)**. The wastewater-based model which was trained iteratively meant that the model training window expands over time, to add one additional training round, for each individual testing round. The intuition was to assess how well a wastewater-based model could estimate SARS-CoV-2 prevalence for a relatively short period of time, such as temporarily between rounds of a prevalence survey. On the other hand, the wastewater-based model which made multi-step prevalence estimates had a fixed training set (of rounds 3 to 16) across the three testing rounds and wastewater-model-based prevalence estimates were made for the individual rounds (without any updating of the training set). In each case, the wastewater-model-based estimates for each individual round were out-of-sample in the sense that prevalence data was omitted for that individual round (and all subsequent rounds). The wastewater-based (gradient boosting) models were trained at an LTLA level, and regional-level estimates were obtained by weighting the LTLA-level estimates by their corresponding populations. Here, accuracy metrics have the interpretation that they measure the predictive performance over time (i.e. three rounds) across all $n^{(obs)}$ observations. *Change Detection* indicates the mean directional accuracy, whilst the corresponding 95% CI is attained by Clopper-Pearson method, otherwise known as the Exact Confidence Interval (1). r denotes the Pearson's correlation between all wastewater-model-based estimates and REACT-1 SARS-CoV-2 prevalence. *MAE* represents the mean absolute error between all model-based predictions and the corresponding LTLA-level (or regional population-weighted average) REACT-1 prevalence estimates. *Mean Prevalence* is the mean average of the LTLA-level (regional population-weighted) REACT-1 prevalence estimates, and is cited as a guide for appraisal of MAE across the rounds. $n^{(geo)}$ denotes the number of geographies covered by both the wastewater surveillance and community prevalence surveys. Median r (Q1, Q3) denotes the median of the LTLA-level/regional Pearson's correlations between their wastewater-model-based estimates and their corresponding REACT-1 SARS-CoV-2 prevalence. This summary statistic may be useful for understanding how well our wastewater model could track the prevalence trends of individual LTLAs (or regions) for these short periods of time of up to month (and therefore, potentially in between rounds of a community prevalence survey). Results for both modelling environments are visualised at a regional level are visualised in Figure 2.

Type	Test Rounds	Training Rounds	Resolution	$n^{(obs)}$	Change Detection (95% CI)	r	MAE	Mean Prevalence	$n^{(geo)}$	Median r (Q1,Q3)
Iterative	17, 18, 19	3-16, 3-17, 3-18	LTLA	1545	75.9% (73.1%,78.7%)	0.60	2.22%	4.5%	309	0.89 (0.53, 0.98)
Multi-Step	17, 18, 19	3-16	LTLA	1545	52.0% (48.7%,55.3%)	0.23	3.08%	4.5%	309	0.62 (0.08, 0.91)
Iterative	17, 18, 19	3-16, 3-17, 3-18	Regional	27	81.5% (61.9%,93.7%)	0.74	1.48%	4.57%	9	0.85 (0.59, 0.99)
Multi-Step	17, 18, 19	3-16	Regional	27	48.1% (28.7%,68.1%)	0.39	3.09%	4.57%	9	0.88 (0.57, 0.93)

Supplementary Material 4 Complementary Use of WBE

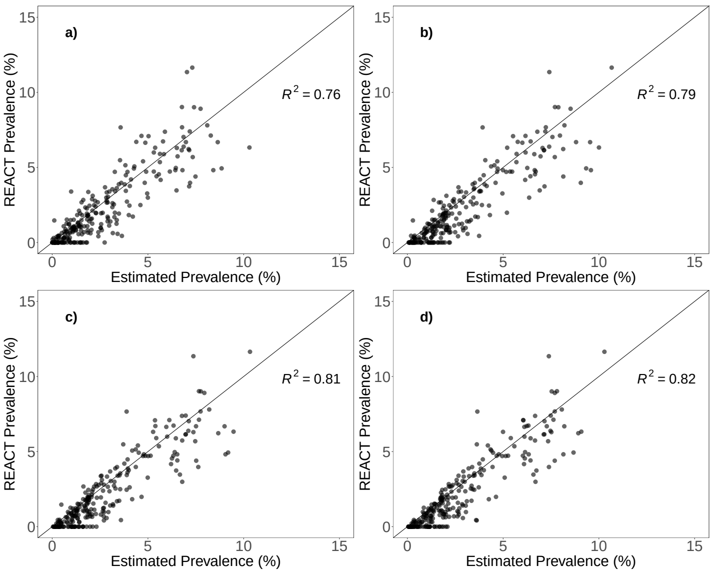

Figure SI 5: **Wastewater-model-based prevalence estimates using varying training set sizes.** From plots a) to d), across rounds 12 to 19 (from 20 May 2021 to 31 March 2022), training set sizes are 40%, 60%, 80%, and 90% respectively per each round's total number of LTLAs. The plot above is for a randomly-selected fold amongst the 50 folds considered in our replicated procedure for training-testing split proportions, and wastewater-model-based prevalence estimates of a fixed test set improve marginally as more training observations are used to calibrate the model. Nevertheless, with just 40% survey coverage, wastewater-model-based estimates remain largely representative of underlying prevalence.

Table SI 9: **Comparison of wastewater-model-based prevalence estimates in the *early* period of rounds 3 to 11 (24 July 2020 to 3 May 2021) using varying training test set sizes**. Wastewater-based gradient boosting models are trained using 40%-90% of each round's observations and a fixed 10% of observations per round are used for test set prevalence estimation. The average prevalence across the 50 folds of test sets is 0.54%.

Training-Testing	MAE	r	Change Detection
40% - 10%	0.34 %	0.74	74.57%
60% - 10%	0.33 %	0.74	75.14%
80% - 10%	0.32 %	0.76	76.30%
90% - 10%	0.32 %	0.75	74.57%

Supplementary Material 5 Population-level faecal shedding

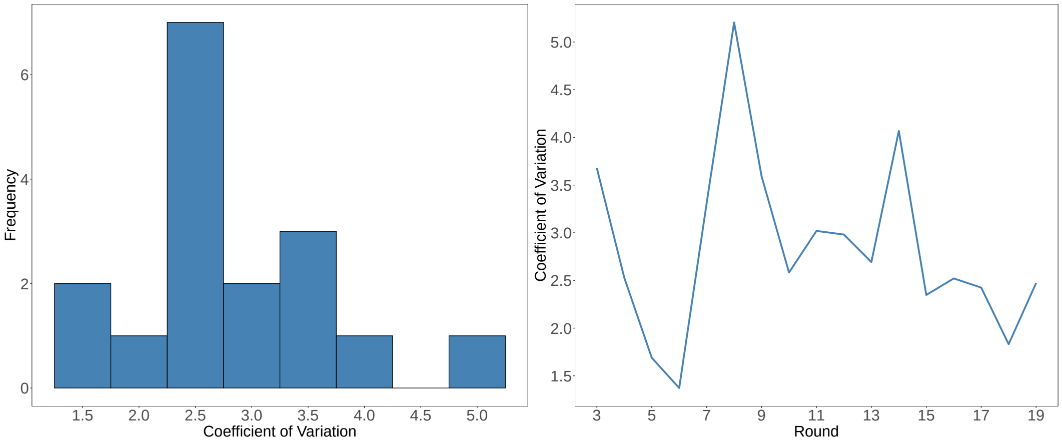

Figure SI 6: **Spatial variability of faecal shedding by round.** Visualisations demonstrate the spatial consistency of the prevalence-to-wastewater relationship. The histogram (left) of the coefficient of variation (CV) values demonstrate that extreme large values of the measure of dispersion are not common, whilst the line plot (right) displays the spatial consistency over time.

Supplementary Material 6 Geospatial Population Estimates (GPEs)

The following section provides further detail regarding the derivation and uncertainty of the GPEs used for our geospatial framework and associated analyses.

Let a_{ij} be the intersection area between the catchment area of STW i and LSOA j. Then, the LSOA area serviced by any of the STWs i is:

$$A_j = \bigcup_i a_{ij}$$

Let p_j be the Office for National Statistics (ONS) 2019 mid-year population estimate for LSOA j. Our geospatial population estimate (GPE) for the intersection area between the catchment area of STW i and the area of LSOA j is

$$\widehat{g_{ij}} = \frac{a_{ij}}{A_j} p_j$$

Thus, for LSOA j, our estimate of the LSOA population serviced is:

$$\widehat{p_j} = \sum_i \widehat{g_{ij}}$$

Summing over all LSOA geographies within an LTLA k, we attain our GPE $\widehat{P_k}$ for each LTLA:

$$\widehat{P_k} = \sum_{j=1}^{J} \widehat{p_j}$$

The GPE for each LTLA enables our mapping of concentrations from an STW level to an LTLA level.

Our approach of using GPEs represents a predictive approach for aligning WBE with the geographies commonly used by public health authorities (2). Consistent with the reported EMHP population coverage estimates (3), we estimate that the wastewater programme had an estimated 74% nationwide testing coverage by its conclusion in March 2022, with a median percentage (of population) sampled for LTLAs of 76%.

The developed approach could, in theory, be applicable to further population-level wastewater monitoring and analyses which require alignment of wastewater catchments to geographies used by public health authorities and community studies. Nevertheless, limitations exist with our geospatial approach. First, the GPEs do not account for the presence of transient and non-resident LTLA populations, which could impact on the wastewater concentrations measured. The transience issue is likely to be more pronounced in the late rounds of the REACT-1 analysis, where community lockdowns and other non-pharmaceutical interventions are relaxed. Second, we recognise the uncertainty surrounding the mid-year population estimates reported by the ONS for LSOAs, which are made for small geographic areas.

Furthermore, potential limitations may yield unrepresentative population estimates for urban centers and/or for geographies which are influenced by time-varying factors such as transient/commuting populations or industrial and agricultural discharges. In theory, such factors are controlled via our flow-normalised wastewater concentrations yet cannot be taken into account when we weight the contribution of each STW to an LTLA based on time-invariant population estimates). The issue of time-varying relationships is likely to depend upon the type and stringency level of simultaneously active non-pharmaceutical interventions.

Further challenges imposed by the EMHP wastewater surveillance programme include the locations of the 302 STWs. Whilst the sampled treatment plants were selected to maximise nationwide coverage and representativeness across England, our GPEs (for each LTLA) indicate a highly positively skewed distribution for the estimated proportions of individual LTLA populations that are sampled within the EMHP programme (Figure SI 7). The skewness in the distribution of estimated proportions of populations sampled is a likely consequence of the usage of intersection areas in our GPEs. We estimate the median LTLA-level proportion of population sampled to be approximately 76%, yet Horesham LTLA, for instance, has an estimated testing coverage of only 2.9% based on the GPE, consistent with the EMHP testing coverage estimate for Horesham. These few outliers can potentially impact on how representative our wastewater concentrations are when mapped to LTLAs with such low testing coverage, and hence, when relating our wastewater data to LTLA-level survey-based prevalence estimates.

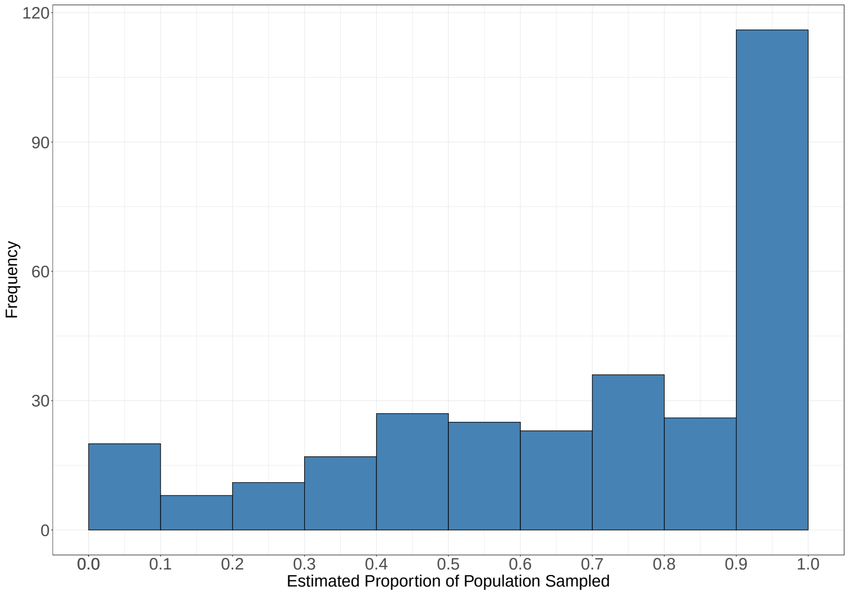

Figure SI 7: **LTLA-level wastewater sampling coverage estimates.** The estimated proportions of LTLA populations that are covered by the EMHP wastewater surveillance programme. The proportions are attained by dividing the LTLA-level GPE by the corresponding ONS LTLA population approximation.

Supplementary Material 7 Further Details of REACT-1

The REACT-1 study was described briefly in Materials and Methods. Here, we outline further details of the study's design and statistical analyses.

Study design

Throughout the 19 distinct rounds, individuals were sampled from the National Health Service (NHS) general practitioner list of patients in England. The list provides details such as name, month and year of birth, gender, and address. To achieve the study's targeted within-round sample size (of between 100,000 and 150,000), up to 750,000 individuals above the age of five years old were random sampled from the general practitioner list and the study team (researchers from Imperial College London and its logistics partner Ipsos) sent personalised invitations to individuals (by mail). Potential participants were provided with study details and asked to indicate their willingness to participate (either via an online portal or via telephone). The participants were provided with both written and video instructions for completing the throat-and-nose swab. If participants provided a swab, they then completed a detailed questionnaire which included questions such as household composition, contact with people known or suspected to have COVID-19, and vaccination history (4, 5, 6).

In terms of the sampling strategy, from round 1 (1 May 2020 to 1 June 2020) to round 12 (20 May 2021 to 7 June 2021), the objective was to obtain LTLA-stratified random samples with approximately equal numbers of participants in each of the 315 LTLAs. However, from round 12 (20 May 2021 to 7 June 2021) to round 19 (8 March 2022 to 31 March 2022), the sampling procedure was adjusted to sample randomly in proportion to LTLA-level populations and thus, correct for under-representation in urban and inner-city areas (relative to more rural and sparsely populated areas) (4).

From round 1 (1 May 2020 to 1 June 2020) to round 13 (24 June 2021 to 12 July 2021), dry swab samples were delivered to the laboratory by a courier on a cold chain. In round 14 (9 September 2021 to 27 September 2021), the study changed to usage of wet (saline) swabs which were sent to the laboratory either by courier (without a cold chain) or priority post. From round 15 (19 October 2021 to 5 November 2021) to round 19 (8 March 2022 to 31 March 2022), the swabs were sent by priority post only. Due to postal-related delays for swabs, the study included a small number of samples obtained after the specified closing date for rounds 14 to 18 (4, 5, 6).

Statistical analysis

The random iterative method (RIM) weighting aimed to correct for the non-response bias in (unweighted) prevalence estimates (7). In particular, to produce the LTLA-level weighted prevalence estimates, the analysis considered varying non-response in varying demographic groups/categories, which included age-sex categories, deprivation index deciles, ethnicity, and LTLA-level populations.

The RIM weighting involved two steps. In the first step, each sample was weighted to LTLA counts and age-sex categories, and the second step adjusted the RIM weighting for all four of the weighting variables. Extreme weights were dampened by trimming the adjustment factor between the first and second step weights at the 1st and 99th percentiles. Thus, the final weights were the first-stage weights multiplied by the trimmed adjustment factor for the second stage (4).

Supplementary Material 8 Wastewater Sources of Uncertainty

Sources of wastewater measurement uncertainty include the sample volume being too low to enable adequate analysis, temperature and time-induced decay, inherent variability of wastewater (possibly due to dilution effects of precipitation), and usage of the Theoretical Limit of Detection (TLoD), below which the concentration cannot be reliably measured. The EMHP surveillance programme aimed to address several of these measurement uncertainties via the adjustment/normalisation of concentrations to account for flow, and by sampling mid-stream during peak load times. Similarly, 3 to 4 samples were taken weekly (at each STW) due to the variability and presence of outliers in detected wastewater signals (3, 8).

Supplementary Material 9 Sensitivity of Geospatial Mapping Approach

Table SI 10: **Sensitivity analysis for geospatial mapping**. Table depicts a diminished relationship between wastewater concentrations and SARS-CoV-2 prevalence, when concentrations from rounds of the REACT-1 are shifted by a lead times of up to six days. Overall correlation measures the correlation between LTLA-level estimated wastewater concentrations and SARS-CoV-2 prevalence. Mean LTLA concentration correlation is the mean of all the LTLA's individual correlations between estimated wastewater concentrations and SARS-CoV-2, whilst the mean LTLA interaction correlation (applicable for rounds 12 to 19) is the average of the LTLA's individual correlation between our proposed vaccination-log concentration interaction and SARS-CoV-2 prevalence.

**Lead Time (Days)**	**Overall Correlation**	**Mean LTLA Concentration Correlation**	**Mean LTLA Interaction Concentration Correlation**
0	0.35	0.39	0.75
1	0.32	0.33	0.75
2	0.29	0.31	0.75
3	0.28	0.30	0.75
4	0.28	0.25	0.74
5	0.27	0.25	0.74
6	0.19	0.16	0.66

Supplementary Material 10 Bayesian Hierarchical Modelling

To ensure robustness of inferences and extend our analysis beyond a discriminative model, within the Bayesian modelling paradigm, we fitted a hierarchical model, which is a generative model type which enables regularisation and spatial effects. Our hierarchical model is fit to training periods using Stan v2.31, which employs Hamiltonian Monte Carlo sampling, a variant of Markov Chain Monte Carlo (MCMC) (9).

Let y_{it} be the REACT-1 weighted prevalence estimate for LTLA i at time/round t. Then, our varying effects Bayesian hierarchical model is as follows:

$$\begin{aligned}
y_{it} &\sim \text{Normal}(\mu_{it}, \sigma^2) \\
\mu_{it} &= \beta_0 + \beta_{LTLA[i]} * C_{it} + \beta_{R[i]} * C_{it} + \beta_{NN[i]} * NC_{it} \\
\beta_0 &\sim \text{Normal}(0, 5) \\
\beta_{LTLA} &\sim \text{Normal}(0, \sigma_{LTLA}^2) \\
\sigma_{LTLA} &\sim \text{InvGamma}(5, 5) \\
\beta_R &\sim \text{Normal}(0, \sigma_R^2) \\
\sigma_R &\sim \text{InvGamma}(5, 5) \\
\beta_{NN} &\sim \text{Normal}(0, \sigma_{NN}) \\
\sigma_{NN} &\sim \text{InvGamma}(5, 5) \\
\sigma &\sim \text{Half-Cauchy}(0, 1)
\end{aligned}$$

where C_{it} denotes the concentration of LTLA i at time/round t, and NC_{it} denotes the weighted average concentration for the neighbours of LTLA i at time/round t. μ and σ represent the mean and standard deviation of the assumed Normal distribution. β_0 is the baseline prevalence. β_{LTLA}, β_R, and β_{NN}, are parameter vectors corresponding to the LTLA-specific, region-specific, and nearest-neighbour specific effects of concentration on the response of prevalence. σ_{LTLA}, σ_R, and σ_{NN} represent the corresponding standard deviations. Above, we employ an index-variable approach within the linear predictor. For example $\beta_{R[i]}$ maps the LTLA i to its corresponding parameter within the region-effect concentration vector. The intuition for spatially-varying effects is that in-sewer network characteristics, treatment plant-level, and spatial correlations between geographies could influence similarities and differences of both concentrations and prevalence levels. For the *late* period of the REACT-1 analysis, the vaccination-log concentration interaction (described in Table SI 2) takes the place of each of the unadjusted wastewater concentrations.

Throughout the analysis, all covariates are standardised such that they take values in $[-1, 1]$. Standardisation facilitates the above usage of conventional, weakly informative prior distributions throughout our model fitting, and assists in ensuring efficient HMC sampling.

Similar to the gradient boosting setup, the model was trained using several calibration rounds from REACT-1, and its predictive performance was estimated out-of-sample using one or more rounds of REACT-1. Our model selection procedure involved consideration of the scientific model's structure, posterior predictive checks, model convergence diagnostics, and estimated out-of-sample predictive accuracy (via the ELPD, expected log pointwise predictive density, from the `loo` package).

In terms of comparing predictive performance to our gradient boosting model, we generate posterior predictions of the response y_{it}. In particular, we use the posterior samples for each observation, and we adopt a conventional approach of using the posterior median of these samples as our best estimate (for each observation). We quantify the uncertainty in our inferences by deriving credible intervals (by taking specified upper and lower quantiles of these samples). Subsequently, metrics such as MAE, for example, are computed as the average absolute difference between the observed prevalence levels and the posterior median estimates.

Throughout both study periods, our best-performing Bayesian hierarchical model did not provide additional predictive accuracy in terms of out-of-sample predictive accuracy. Superior accuracy of the gradient boosting model may be a consequence of the highly flexible and predictive nature of the modern gradient boosting algorithm (of `xgboost`) which readily handles time-varying non-linearities. Nevertheless, the additional modelling investigation enabled a further robustness check of the inferences drawn regarding the reliability of wastewater-based modelling.

Supplementary Material 11 Vaccination-Log Concentration Interaction Variable

In terms of the statistical controls in our analysis, there exist obvious limitations with our proposed usage of a simple vaccination-concentration interaction. Aside from the potential differential impacts of vaccination on distinct demographies, the proposed interaction variable would only remain (possibly) valid for a restricted time period due to impacts of waning vaccine-induced immunity. We further do not account for the highly complex nature of naturally-acquired immunity. The interaction variable's usage is additionally challenged by the disparate impact of different variants (and sub-variants) on the likelihood of SARS-CoV-2 reinfection. Importantly, we do not draw *any causal conclusions* regarding the impact of vaccination on faecal shedding due to the potential presence of confounding. Indeed, we emphasise, that strong temporal correlation is not necessarily indicative of a direct (or causal) relationship with prevalence, as the directional association may be an artefact of numerous epidemic conditions. Complex, concurrent epidemic characteristics include a monontonically increasing vaccination proportion, similarly rising prevalence levels, waning immunity (either vaccine-induced or naturally-acquired), reduced immunity to particular new variants, and other possibly unobserved confounders.

Supplementary Material 12 Acronyms

Table SI 11: **Acronyms used throughout this paper.**

Variable	Meaning	Details
95% CrI	95% Credible Interval	An unobserved parameter lies in this interval with a specified 95% probability.
EMHP	Environmental Monitoring for Health Protection	The EMHP wastewater surveillance programme tested untreated sewage across England for fragments of SARS-CoV-2. The objectives of the programme were to monitor wastewater viral concentrations of SARS-CoV-2 RNA, variants of concern (VOC), and variants under investigation (VUI).
gc/l	Gene copies per litre (gc/l)	The reported measuresments of wastewater concentrations obtained via RT-qPCR and flow normalisation.
GPE	Geospatial population estimate	Our population estimates for geographies based on a combination of intersection (spatial) areas and reported population estimates for small geographies.
LSOA	Lower Layer Super Output Area	Small regional geographies in England which combine to form an LTLA.
LTLA	Lower Tier Local Authority	LTLA-level wastewater measurements per round. These are obtained via the weighted contribution of each treatment plant's average concentration for the round (described in Materials and Methods).
MAE	Mean Absolute Error	The mean average of the absolute difference between the wastewater-model-based estimates and the REACT-1 prevalence levels.
MCMC	Markov Chain Monte Carlo	A family of sampling algorithms which employs the theory of Markov Chains to sample a random variable.
NHS	National Health Service	The public healthcare system in the United Kingdom.
NPI	Non-pharmaceutical intervention	In the context of the COVID-19 pandemic, these are public health measures, excluding medication-based measures, taken to control transmission of the SARS-CoV-2.
ONS	Office for National Statistics	An independent producer of national statistics across the UK.
PI	Prediction Interval	We use 95% Prediction Intervals for our wastewater-based (gradient boosting) model's prevalence estimates which were estimated by 5,000 non-parametric bootstrap replicates of training datasets.
REACT-1	Real-time Assessment of Community Transmission	The REACT-1 programme was initiated in May 2020 with an objective of tracking the spread of SARS-CoV-2 across communities in England. Across 19 distinct rounds of cross-sectional surveys, random samples of the English population (over 5 years of age) were taken. Rounds lasted between 15 and 31 days, and the programme concluded in March 2022.
RNA	Ribonucleic acid	RNA is a nucleic acid present in all living cells.
RT-PCR	Reverse Transcriptase Polymerase Chain Reaction	The commercial laboratory of the REACT-1 study used RT-PCR. The method combines reverse transcription of RNA into DNA and amplification of specific DNA targets using polymerase chain reaction.
RT-qPCR	Reverse Transcriptase quantitative Polymerase Chain Reaction	The quantification method is described in (10). Briefly, RT-qPCR combines reverse transcription and quantitative PCR, with the aim of reducing inhibition in RNA.
STW	Sewage treatment works	STWs are treatment plants which typically service extensive urban areas (like towns and cities).
TLoD	Theoretical limit of detection	The wastewater concentration (160 gc/l) below which the EMHP estimate that concentration cannot be reliably estimated.
UKHSA	United Kingdom Health Security Agency	A nationwide organisation in the United Kingdom which assumes responsibility for public health protection.
VOC	Variants of Concern	SARS-CoV-2 variants that were highlighted by the World Health Organisation (WHO) to be particularly dangerous in terms of increased transmissibility.
VUI	Variants under investigation	SARS-CoV-2 variants were being tracked by the EMHP wastewater surveillance programme.
WBE	Wastewater-based epidemiology	WBE involves collection of urine and stool samples from sewage treatment works (STWs). By subsequently incorporating factors such as daily flow rates, human excretion rates, and STW catchment population sizes, per-capita consumption, use, or exposure can be obtained.
WHO	World Health Organisation	An agency of the United Nations which aims to improve international public health.

Supplementary Material 13 Timeline of REACT-1 and Estimated Number of LTLAs in EMHP Programme

Table SI 12: Dates (DD/MM/YYYY) for individual rounds of the REACT-1 study and the corresponding estimated number of LTLAs which were mapped to STWs which reported measurements within the corresponding time intervals. Our overall studied period, where the EMHP wastewater surveillance programme coincides with the REACT-1 study, covers from rounds 3 to 19 (from 24 July 2020 to 31 March 2022).

Round Number	Round Start Date	Round End Date	Number of LTLAs
1	01/05/2020	01/06/2020	-
2	19/06/2020	07/07/2020	-
3	24/07/2020	11/08/2020	145
4	20/08/2020	08/09/2020	145
5	18/09/2020	05/10/2020	146
6	16/10/2020	02/11/2020	146
7	13/11/2020	03/12/2020	146
8	06/01/2021	22/01/2021	146
9	04/02/2021	23/02/2021	233
10	11/03/2021	30/03/2021	299
11	15/04/2021	03/05/2021	301
12	20/05/2021	07/06/2021	303
13	24/06/2021	12/07/2021	306
14	09/09/2021	27/09/2021	307
15	19/10/2021	05/11/2021	309
16	23/11/2021	14/12/2021	309
17	05/01/2022	20/01/2022	309
18	08/02/2022	01/03/2022	309
19	08/03/2022	31/03/2022	309

Supplementary Material 14 Wastewater Variant Detections

From a public health perspective and for our analysis, the ability to track variants is important to understand the relationship between wastewater concentrations and our community prevalence estimates. Specifically, the EMHP programme raised the conjecture of an Omicron effect which results in lower viral faceal shedding and substantially alters the relationship between wastewater signals and clinical measures (3). The variant detections were attained via genomic sequencing of wastewater samples from both STWs and sewer network sites across England, and detections were reported as either a confirmed or a possible status. More recently, the importance of accounting for the predominant variant was demonstrated by research of clinical cases showing that Omicron infections yielded the lowest community-level SARS-CoV-2 waste shedding rates, compared to the early/parental SARS-CoV-2 and Delta variant (11). Within our analysis, we have documented the apparent reduced population-level faecal shedding induced by the onset of the Omicron BA.1 and BA.2 sub-variants.

Supplementary Material 15 Further Results

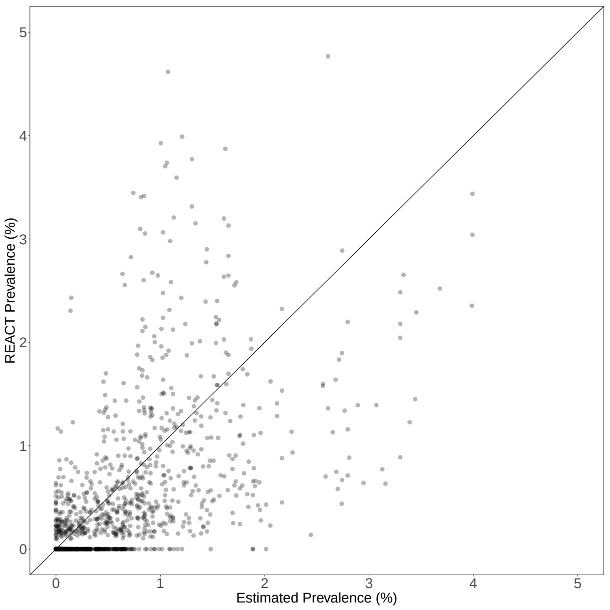

Figure SI 8: **Iteratively-updating wastewater-model-based prevalence estimates for rounds 7 to 11 (13 November 2020 to 3 May 2021).** Visualisation depicts REACT-1 prevalence versus wastewater-model-based estimates for the corresponding round. The wastewater-based model was trained iteratively - in the sense that the model training window expands over time, to add one additional training round, for each individual testing round. The intuition is to assess how well a wastewater-based model could estimate SARS-CoV-2 prevalence for a relatively short period of time, such as temporarily between rounds of a prevalence survey.

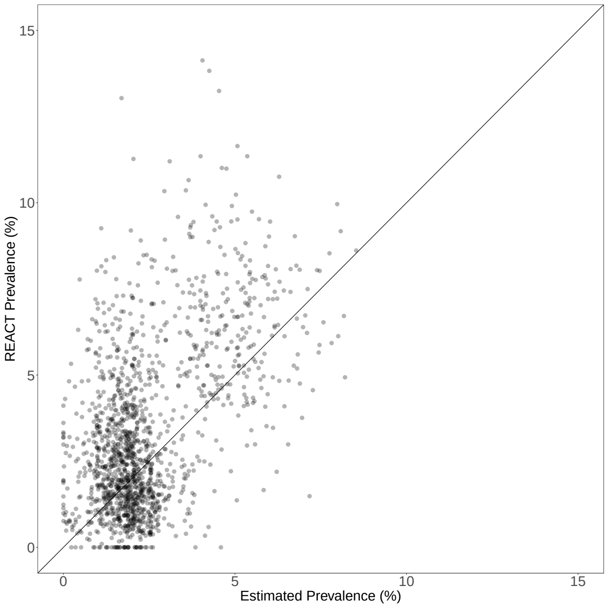

Figure SI 9: **Iteratively-updating wastewater-model-based prevalence estimates for rounds 15 to 19 (19 October 2021 to 31 March 2022).** Visualisation depicts REACT-1 prevalence versus wastewater-model-based estimates for the corresponding round. The wastewater-based model was trained iteratively - in the sense that the model training window expands over time, to add one additional training round, for each individual testing round. The intuition is to assess how well a wastewater-based model could estimate SARS-CoV-2 prevalence for a relatively short period of time, such as temporarily between rounds of a prevalence survey.

References

[1] C. J. Clopper and E. S. Pearson. The Use of Confidence or Fiducial Limits Illustrated in the Case of the Binomial. *Biometrika*, 26(4):404–413, 1934.

[2] Till Hoffmann, Sarah Bunney, Barbara Kasprzyk-Hordern, and Andrew C. Singer. Wastewater catchment areas in Great Britain. November 2022.

[3] UKHSA. EMHP wastewater monitoring of SARS-CoV-2 in England: 15 July 2020 to 30 March 2022, May 2022.

[4] Paul Elliott, Matthew Whitaker, David Tang, Oliver Eales, Nicholas Steyn, Barbara Bodinier, Haowei Wang, Joshua Elliott, Christina Atchison, Deborah Ashby, Wendy Barclay, Graham Taylor, Ara Darzi, Graham S. Cooke, Helen Ward, Christl A. Donnelly, Steven Riley, and Marc Chadeau-Hyam. Design and Implementation of a National SARS-CoV-2 Monitoring Program in England: REACT-1 Study. *American Journal of Public Health*, 113(5):545–554, May 2023.

[5] Paul Elliott, Oliver Eales, Nicholas Steyn, David Tang, Barbara Bodinier, Haowei Wang, Joshua Elliott, Matthew Whitaker, Christina Atchison, Peter J. Diggle, Andrew J. Page, Alexander J. Trotter, Deborah Ashby, Wendy Barclay, Graham Taylor, Helen Ward, Ara Darzi, Graham S. Cooke, Christl A. Donnelly, and Marc Chadeau-Hyam. Twin peaks: The Omicron SARS-CoV-2 BA.1 and BA.2 epidemics in England. *Science*, 376(6600):eabq4411, June 2022.

[6] Paul Elliott, David Haw, Haowei Wang, Oliver Eales, Caroline E. Walters, Kylie E. C. Ainslie, Christina Atchison, Claudio Fronterre, Peter J. Diggle, Andrew J. Page, Alexander J. Trotter, Sophie J. Prosolek, The COVID-19 Genomics UK (COG-UK) Consortium, Deborah Ashby, Christl A. Donnelly, Wendy Barclay, Graham Taylor, Graham Cooke, Helen Ward, Ara Darzi, and Steven Riley. REACT-1 round 13 final report: exponential growth, high prevalence of SARS-CoV-2 and vaccine effectiveness associated with Delta variant in England during May to July 2021. preprint, Epidemiology, September 2021.

[7] Trevor Sharot. Weighting survey results. *J Mark Res Soc*, 28(3):269–284, 1986.

[8] Matthew J. Wade, Anna Lo Jacomo, Elena Armenise, Mathew R. Brown, Joshua T. Bunce, Graeme J. Cameron, Zhou Fang, Kata Farkas, Deidre F. Gilpin, David W. Graham, Jasmine M.S. Grimsley, Alwyn Hart, Till Hoffmann, Katherine J. Jackson, David L. Jones, Chris J. Lilley, John W. McGrath, Jennifer M. McKinley, Cormac McSparron, Behnam F. Nejad, Mario Morvan, Marcos Quintela-Baluja, Adrian M.I. Roberts, Andrew C. Singer, Célia Souque, Vanessa L. Speight, Chris Sweetapple, David Walker, Glenn Watts, Andrew Weightman, and Barbara Kasprzyk-Hordern. Understanding and managing uncertainty and variability for wastewater monitoring beyond the pandemic: Lessons learned from the United Kingdom national COVID-19 surveillance programmes. *Journal of Hazardous Materials*, 424:127456, February 2022.

[9] {Stan Development Team}. Stan Modeling Language Users Guide 2023 and Reference Manual, 2.3.1. https://mc-stan.org.

[10] Luke S. Hillary, Kata Farkas, Kathryn H. Maher, Anita Lucaci, Jamie Thorpe, Marco A. Distaso, William H. Gaze, Steve Paterson, Terry Burke, Thomas R. Connor, James E. McDonald, Shelagh K. Malham, and David L. Jones. Monitoring SARS-CoV-2 in municipal wastewater to evaluate the success of lockdown measures for controlling COVID-19 in the UK. *Water Research*, 200:117214, July 2021.

[11] Sarah M. Prasek, Ian L. Pepper, Gabriel K. Innes, Stephanie Slinski, Walter Q. Betancourt, Aidan R. Foster, Hayley D. Yaglom, W. Tanner Porter, David M. Engelthaler, and Bradley W. Schmitz. Variant-specific SARS-CoV-2 shedding rates in wastewater. *Science of The Total Environment*, 857:159165, January 2023.
